# Taxonomical review of *Prosymnaangolensis* Boulenger, 1915 (Elapoidea, Prosymnidae) with the description of two new species

**DOI:** 10.3897/zookeys.1121.85693

**Published:** 2022-09-15

**Authors:** Werner Conradie, Chad Keates, Ninda L. Baptista, Javier Lobón-Rovira

**Affiliations:** 1 Port Elizabeth Museum (Bayworld), P.O. Box 13147, Humewood 6013, Gqeberha, South Africa Nelson Mandela University George South Africa; 2 Department of Nature Conservation Management, Natural Resource Science and Management Cluster, Faculty of Science, George Campus, Nelson Mandela University, George, South Africa Port Elizabeth Museum (Bayworld) Gqeberha South Africa; 3 National Geographic Okavango Wilderness Project, Wild Bird Trust, Sandton, South Africa National Geographic Okavango Wilderness Project, Wild Bird Trust Sandton South Africa; 4 South African Institute for Aquatic Biodiversity (SAIAB), Makhanda, South Africa South African Institute for Aquatic Biodiversity (SAIAB) Makhanda South Africa; 5 Department of Zoology and Entomology, Rhodes University, Makhanda, South Africa Rhodes University Makhanda South Africa; 6 Instituto Superior de Ciências da Educação da Huíla (ISCED-Huíla), Rua Sarmento Rodrigues, Lubango, Angola Universidade do Porto Vairao Portugal; 7 CIBIO-InBIO, Centro de Investigação em Biodiversidade e Recursos Genéticos, Laboratório Associado, Universidade do Porto, Campus Agrário de Vairão, Rua Padre Armando Quintas, 4485-661 Vairão, Portugal Universidade do Porto Porto Portugal; 8 Departamento de Biologia, Faculdade de Ciências, Universidade do Porto, 4099-002 Porto, Portugal Instituto Superior de Ciências da Educação da Huíla (ISCED-Huíla), Rua Sarmento Rodrigues Lubango Angola; 9 BIOPOLIS Program in Genomics, Biodiversity and Land Planning, CIBIO, Campus de Vairão, 4485-661 Vairão, Portugal Biodiversity and Land Planning, CIBIO Vairao Portugal

**Keywords:** Africa, Angola, cryptic species, fossorial, Kalahari, Serpentes

## Abstract

African Shovel-snout snakes (*Prosymna* Gray, 1849) are small, semi-fossorial snakes with a unique compressed and beak-like snout. *Prosymna* occur mainly in the savanna of sub-Saharan Africa. Of the 16 currently recognised species, four occur in Angola: *Prosymnaambigua* Bocage, 1873, *P.angolensis* Boulenger, 1915, *P.frontalis* (Peters, 1867), and *P.visseri* FitzSimons, 1959. The taxonomical status and evolutionary relationships of *P.angolensis* have never been assessed due to the lack of genetic material. This species is known to occur from western Angola southwards to Namibia, and eastwards to Zambia, Botswana and Zimbabwe. The species shows considerable variation in dorsal colouration across its range, and with the lower ventral scales count, an ‘eastern race’ was suggested. In recent years, *Prosymna* material from different parts of Angola has been collected, and with phylogenetic analysis and High Resolution X-ray Computed Tomography, the taxonomic status of these populations can be reviewed. Strong phylogenetic evidence was found to include the *angolensis* subgroup as part of the larger *sundevalli* group, and the existence of three phylogenetic lineages within the *angolensis* subgroup were identified, which each exhibit clear morphological and colouration differences. One of these lineages is assigned to the nominotypical *P.angolensis* and the other two described as new species, one of which corroborates the distinct eastern population previously detected. These results reinforce that a considerable part of Angolan herpetological diversity is still to be described and the need for further studies.

## Abstract (pt)

As cobras-de-focinho-de-pá africanas (*Prosymna*) são pequenas cobras semi-fossoriais com um focinho único, achatado e em forma de bico, que ocorrem principalmente na savana da África subsaariana. Das 16 espécies actualmente reconhecidas, quatro existem em Angola: *Prosymnaambigua* Bocage, 1873, *P.angolensis* Boulenger, 1915, *P.frontalis* (Peters, 1867), e *P.visseri* FitzSimons, 1959. O estatuto taxonómico e as relações evolutivas de *P.angolensis* nunca foram avaliados devido à falta de material genético. A espécie ocorre desde o oeste de Angola, para sul até a Namíbia, e para este em direcção à Zâmbia, Botswana e Zimbábue. Na sua área de ocorrência, esta espécie tem variação principalmente na coloração dorsal, e com base no menor número de escamas ventrais, foi sugerida a existência de uma raça oriental. Recentemente foi amostrado material de *Prosymna* de várias partes de Angola, e com recurso a análises filogenéticas e a tomografia computadorizada de raios-X de alta resolução, foi possível rever o estatuto taxonómico destas populações. Encontrámos fortes evidências filogenéticas para incluir o subgrupo *angolensis* como parte do grupo *sundevalli*. Revelámos a existência de três linhagens filogenéticas no subgrupo *angolensis*. Atribuímos uma dessas linhagens ao *P.angolensis* nominotípico, e descrevemos as outras duas como espécies novas, uma das quais corrobora a população oriental previamente detectada. Estes resultados reforçam a ideia de que uma parte considerável da diversidade herpetológica angolana está ainda por descrever, e a necessidade de mais estudos.

## Introduction

African Shovel-snout snakes, belonging to the genus *Prosymna*, are small terrestrial snakes occurring in sub-Saharan Africa, mostly associated with semi-desert, savanna and miombo woodlands (Broadley 1980). They are characterised by a compressed skull with a depressed, upward pointing snout with a sharp horizontal edge, which allows them to live a semi-fossorial lifestyle (Broadley 1990; Branch 1998; Marais 2004; Spawls et al. 2018; Pietersen et al. 2021). The absent or anteriorly reduced dentary teeth, and the unique modified blade-like rear maxillary teeth allow them to slit open soft-shelled reptile eggs, on which they feed almost exclusively, although some species feed on hard-shelled gecko eggs (Broadley 1979, 1980; Heinicke et al. 2020). Due to this unusual skull compression, snout shape and modified maxillary teeth, the higher taxonomical level of these snakes has been in flux. However, modern phylogenetic analyses have allowed them to be assigned to their own family, Prosymnidae (superfamily Elapoidea), sister to the family Psammophiidae (Vidal et al. 2008; Kelly et al. 2009; Pyron et al. 2013; Figueroa et al. 2016; Zaher et al. 2019). Currently, *Prosymna* is represented by 16 species (Heinicke et al. 2020; Uetz et al. 2022), and recent phylogenetic work has shown that cryptic diversity may exist in the genus, especially within *P.ambigua*, *P.frontalis*, and *P.stuhlmanni* (Heinicke et al. 2020).

The first *Prosymna* recorded from Angola were documented by Bocage (1873). He assigned material collected from Mossamedes [= Moçâmedes] and Biballa [= Bibala] to *Prosymnafrontalis* (Peters, 1867) and described *Prosymnaambiguus* Bocage, 1873 (= *P.ambigua*) from Duque de Bragança (= Calandula) based on the higher number of midbody scale rows (17 vs. 15), the shape of the rostral and the larger parietal scales when compared to *P.frontalis*. Later, Bocage (1882) once again noted that the Angolan specimens of *P.frontalis* did not fully agree morphologically with the original description provided by Peters (1867). Afterwards, he provided a more detailed account including additional material collected from Angola, reporting the morphological variation observed when compared to *P.frontalis* types (Bocage 1895). The main confusion that Bocage faced at the time was that the two type specimens (one adult and one juvenile) of *P.frontalis* represented two separate species: *P.frontalis* and *P.bivittata* (see Mertens 1955; Broadley 1980). His material agrees in part with the juvenile specimen (= *P.bivittata*) in the number of postoculars (= 1) and lower subcaudal scale count (< 25), while it differed from the larger specimen (= *P.frontalis* lectotype) in lower subcaudal scale count (50 vs. 17–25) and the number of postorbital scales (one vs. two). The only difference was that the internasal condition (single bandlike scale) of the Angolan material was in agreement with the *P.frontalis* lectotype and not with the juvenile of *P.bivittata*. Due to this confusion in the overlapping morphology with the original *P.frontalis* types, Bocage decided not to take any taxonomic actions and kept referring to material from Angola under the name *P.frontalis*. It should be noted that the Reptile Database (Uetz et al. 2022) states that *P.angolensis* is a *nomen novum* for ‘*P.frontalis* Bocage, 1895’. This is incorrect, as Bocage (1895) never described *P.frontalis* as a new species.

Boulenger (1915), with no proper justification, described the Angolan material previously assigned to *P.frontalis* by Bocage (1873, 1882, 1895) as a new species, i.e., *P.angolensis* Boulenger, 1915, stating only how it differed from the only other Angolan congener at the time, *P.ambigua*. Additional material under the names *P.ambigua* and *P.angolensis* were recorded from central and western Angola by Monard (1931, 1937), Mertens (1937, 1938) and Bogert (1940). Until the mid-19^th^ century, this was the only species of *Prosymna* known to occur in Angola, until Charles Koch collected a specimen from south-western Angola, that was later described as a new species, *Prosymnavisseri* FitzSimons, 1959. When Broadley (1980) reviewed the *Prosymna* genus, he documented nominotypical *P.frontalis* from Benguela, collected by Wulf Haacke in the 1970’s. This brought the number of *Prosymna* species occurring in Angola to four (Branch 2018; Marques et al. 2018).

Over the decades, the relationship of *P.angolensis* to other *Prosymna* species was addressed on a morphological basis by several authors, being considered as more closely related to *P.sundevalli* (Boulenger 1894), *P.lineata* (Bocage 1895), *P.ambigua* (Bogert 1940), and *P.frontalis* (Mertens 1955). Finally, with the whole genus revision, Broadley (1980) was the first to attempt grouping *Prosymna* species based on shared morphological characters. He identified three main species groups (the *ambigua* group = *ambigua*, *ornatissima*, *semifasciata*, *stuhlmanni*; the *meleagris* group = *greigerti*, *meleagris*, *ruspolli*, *somalica*; the *sundevalli* group = *bivittata*, *lineata*, *sundevalli*). Whilst he briefly mentioned it might be part of the *sundevalli* group, he could not confidently assign *P.angolensis* to any of these groupings. With the aid of phylogenetics, Heinicke et al. (2020) partly validated Broadley’s groupings, but due to the lack of *P.angolensis* genetic material, its relationship to its congeners remained unclear.

**Figure 1.**
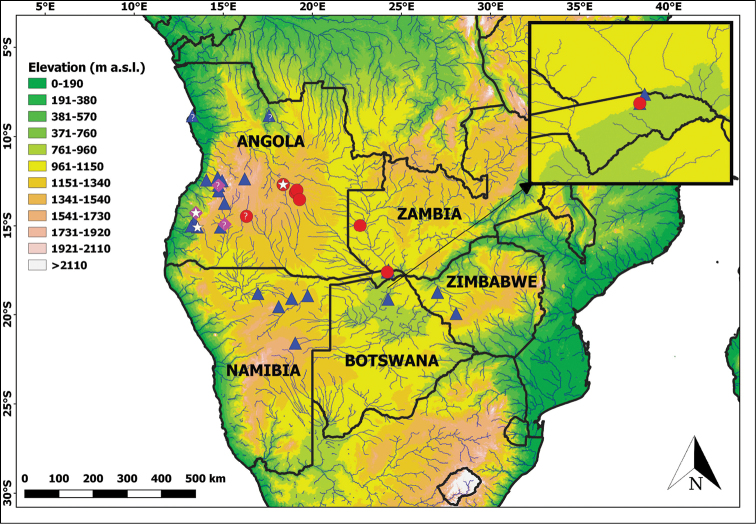
Geographic records of the *Prosymnaangolensis* group, based on all literature records and newly examined material, including nominotypical *P.angolensis* (blue triangles), *Prosymna* ‘Eastern’ = *P.lisima* sp. nov. (red circles) and *Prosymna* ‘Coastal’ = *P.confusa* sp. nov. (purple diamonds). White stars represent respective type localities. Question marks represent material tentatively assigned to that species, but needs confirmation. Blue lines represent major river systems. Top right inset represents the eastern Zambezi Region, to show sympatry between *P.angolensis* and *Prosymna* ‘Eastern’ = *P.lisima* sp. nov.

Since the description of *P.angolensis*, only a handful of specimens have been recorded from Angola (Monard 1931, 1937; Mertens 1937; Bogert 1940; Hellmich 1957; Baptista et al. 2019; Ceríaco et al. 2021a; Conradie et al. 2021). Specimens from outside of Angola were documented from northern Namibia (Mertens 1955), the Zambezi Region (= Caprivi Strip) in north-eastern Namibia, western Zambia, northern Botswana (Broadley 1980), and north-eastern Zimbabwe (Broadley 1995) (see Fig. 1). However, the specimens from Zambia and the Zambezi Region differ from the nominotypical Angolan form in the number of ventral scales (121–129 vs. 134–142), number of postoculars (two vs. one), and dorsal colouration (large black confluent black blotches vs. mostly two dorsal rows of small paired black spots) (Broadley 1980). Interestingly, one specimen from Vila da Ponte (= Kuvango), in west/central Angola, exhibits the same combination of features (lower ventral scale counts, two postoculars and a dorsal pattern consisting of confluent black blotches posteriorly) (Monard 1937 fide Broadley 1980). Despite noticing these differences, Broadley (1980) did not make taxonomic changes, stating that a larger series was needed to clarify this issue.

Recent herpetological surveys undertaken in eastern and southwestern Angola led to the collection of several specimens assigned to *P.angolensis* (Branch 2018; Baptista et al. 2019; Conradie et al. 2021). The first was collected by William R. Branch from the coastal semi-arid lowlands in Namibe Province and was initially assigned to *P.ambigua* (Branch 2018). This identification was problematic because it had no morphological justification and was collected from a locality well outside the known range of that species. Upon re-examination of the specimen, it was concluded that it was a uniformly coloured *P.angolensis* (WC unpubl. data), another problematic identification, as it did not agree with the species description and this was the first record of *P.angolensis* from the more arid coastal plains of Angola. Nominotypical *P.angolensis*, which fully agrees with the original description, was collected from Bicuar National Park in southwestern Angola (Baptista et al. 2019). Finally, during several expeditions to document the biodiversity of the headwaters of the Angolan Okavango-Cuando-Zambezi river basins in east-central Angola, a series of specimens was collected, and tentatively assigned to *P.angolensis* based on external morphology (Conradie et al. 2021), but they exhibited the same distinct characteristics reported from western Zambia and north-eastern Namibia specimens by Broadley (1980). This new material allowed us to revisit the taxonomical status and evolutionary relationships of *P.angolensis*, and investigate the morphological differences observed among these different populations using modern phylogenetic analysis and High Resolution X-ray Computed Tomography (HRCT).

## Materials and methods

### Sampling

At several sites during the 2016–2019 National Geographic Okavango Wilderness Project surveys, standard Y-shape intercept drift fence funnel trap arrays were deployed to passively collect specimens. Each Y-shaped trap array consisted of three drift fences (each 10 m long and 50 cm high) radiating from a central pitfall trap, with six one-way funnel traps placed on adjacent sides of each drift fence and three one-way funnels at the terminal ends of each drift fence. Trap arrays were installed in varied habitats to ensure the highest possible richness of captured species (Conradie et al. 2021). Consequently, a total of eight *Prosymna* individuals were captured. Snakes were euthanized by injecting them subcutaneously with tricaine methane sulfonate (MS222) solution (Conroy et al. 2009), after which they were formalin-fixed for 48 hours and transferred to 70% ethanol for long-term storage. Prior to formalin fixing, tissue samples (liver) were preserved in 99% ethanol for subsequent genetic analysis. In addition, we included a recently collected specimen of nominotypical *P.angolensis* from Bicuar National Park (Baptista et al. 2019) and a specimen that was tentatively assigned to *P.ambigua* from southwestern Angola (Branch 2018), to complement the morphological and genetic analyses. Voucher specimens are held in the herpetological collection of Port Elizabeth Museum at Bayworld Complex (**PEM**), Gqeberha, South Africa and Colecção Herpetológica do Lubango (**CHL**), currently deposited in Instituto Superior de Ciências de Educação da Huíla (**ISCED-Huíla**), Lubango, Angola.

Additionally, *Prosymnaangolensis* material examined by Broadley (1980) from the following institutions was included in this study:

**AMNH**American Museum of Natural History, New York, USA;

**CAS**California Academy of Sciences, Los Angeles, USA;

**MBL** Museu Bocage, Lisbon, Portugal;

**MCZ**Museum Comparative Zoology, Harvard, USA;

**NMW**Naturhistorisches Museum zu Wien, Vienna, Austria;

**NMZB/UM**National Museum of Zimbabwe, Buluwayo, Zimbabwe;

**SAM**South African Museum (now Iziko Museums of South Africa), Cape Town, South Africa;

**SMF**Forschungsinstitut und Natur-Museum Senckenberg, Frankfurt-am-Main, Germany;

**TM**Transvaal Museum (now Ditsong National Museum of Natural History Northern Flagship Institute);

**USBN** National Museum of Natural History, Washington, USA.

The original datasheets were made available to the authors by Sheila Broadley. Photographs of the following material were examined by WC: AMNH R50504, MCZ R-32468, NMW 19275.2 and SAM ZR16574.

### DNA extraction, amplification, and sequencing

A standard salt extraction method (Bruford et al. 1992) was used to isolate DNA from the tissue sample using ATL lysis and AE elution buffers. Standard PCR procedures were utilised to amplify one partial mitochondrial ribosomal gene (ribosomal ribonucleic acid [*16S*]), two partial mitochondrial genes (cytochrome b [*cyt-b*] and NADH-dehydrogenase subunit 2 [*ND2*]) and one partial nuclear gene (oocyte maturation factor [*c-mos*]). The specific primer pairs used can be found in Table 1. Each amplification was conducted with a PCR mixture to the total volume of 25 µl containing 12.5 μl TopTaq Mastermix (Ampliqon; containing 2× master mix, 1.5 mM MgCl_2_, 0.4 mM dNTPs, and Ampliqon Taq DNA polymerase), 2 µl forward primer (10 µM), 2 µl reverse primer (10 µM), 6.5 µl de-nucleated water and 2 µl genomic DNA (20–50 ng/µl). The cycling profile for all the genes was as follows: initial denaturing step at 94 °C for 5 min, followed by 35 cycles of 94 °C for 30 s, 50–60 °C for 45 s, and 72 °C for 45 s, with a final extension at 72 °C for 8 min. The prepared PCR products were purified and sequenced at Macrogen Corp. (Amsterdam, Netherlands) with the forward primers only.

**Table 1.** Primers and PCR protocols used to generate sequences for the study.

Gene	Primer	Source	Annealing temperature (°C)
*16S*	L2510: 5’—CGCCTGTTTATCAAAAACAT-—3’	Palumbi (1996)	50
	H3080: 5’-CCGGTCTGAACTCAGATCACGT-3’		
*cyt-b*	WWF: 5’—AAAYCAYCGTTGTWATTCAACTAC—3’	Whiting et al. (2003)	52
	Cytb‐R2: 5’—GGGTGRAAKGGRATTTTATC—3’		
*ND2*	ND2-F1-METF1: 5’—AAGCTTTCGGGCCCATACC—3’	Heinicke et al. (2014)	56
	ND2-R1-TRPR3: 5’—TTTAGGGCTTTGAAGGC—3’		
*c-mos*	S77: 5’—CAT GGACTGGGATCAGTTATG—3’	Slowinski and Lawson (2002)	52
	S78: 5’—CCTTGGGTGTGATTTTCT CACCT—3’		

### Phylogenetic analyses

The phylogenetic placement of the newly collected *Prosymna* samples were estimated by comparing them with the sequenced data from Heinicke et al. (2020). In addition to the ingroup taxa (12 of the 16 currently recognised *Prosymna* species), the dataset was supplemented with sequences from closely related genera that were obtained from GenBank and used as outgroups (Appendix 1: Table A1).

The sequence trace files were checked using BioEdit Sequence Alignment Editor v. 7.2.5 (Hall 1999) and aligned with accessioned GenBank sequences using MEGA v.6.0 (Tamura et al. 2013) and the ClustalW alignment method. Four individual alignments were created (*16S*, *ND2*, *cyt-b*, *c-mos*) and added to the existing dataset from Heinicke et. al (2020), along with two additional nuclear markers (*RAG1* and *ENC-1*) from the same paper, which were used to resolve the deeper nodes. The congruency of the individual genes was tested using the homogeneity test implemented in PAUP4 v. 4.0a (Swofford 2003). All six gene alignments were not significantly different from one another allowing the creation of a concatenated dataset.

DAMBE v. 6.4.67 (Xia 2013) was used to test for saturation using the individual as well as the combined first and second codon positions of each gene. Saturation was absent from every marker, so a gene-partitioned dataset was created for the phylogenetic reconstruction. The optimal partition scheme and best-fitting models of molecular evolution were selected using ModelFinder implemented in IQ-TREE v.2.1.2 (Minh et al. 2021). The following settings were used: -p partition file (each partition has own evolution rate), a greedy strategy and the FreeRate heterogeneity model excluded (only invariable site and Gamma rate heterogeneity considered) (Chernomor et al. 2016; Kalyaanamoorthy et al. 2017). The best-fitting model scheme selected included the following three partitions and models of evolution: TIM2+I+G (*16S*); GTR+I+G (*cyt-b*, *ND2*); TN+G (*c-mos*, *RAG1*, *ENC-1*). MrBayes v.3.2.7a (Ronquist et al. 2012) and BEAST2 v.2.6.6 (Bouckaert et al. 2019) were not able to implement TIM2 or TN, so the next best alternative (GTR) was used in their place.

### Phylogenetic reconstruction

Maximum likelihood (**ML**) analysis was conducted using IQ-TREE v.2.1.2 (Nguyen et al. 2015). A random starting tree was used using the gene-partitioned scheme mentioned above, the ultrafast bootstrap approximation (**UFBoot**) method (Hoang et al. 2018) and 1000 bootstrap replicates. Bayesian inference (**BI**) analysis was implemented using MrBayes v. 3.2.7a (Ronquist et al. 2012) and BEAST2 v. 2.6.6 (Drummond and Rambaut 2007; Suchard and Rambaut 2009) on the CIPRES Science Gateway XSEDE online resource (http://www.phylo.org; Miller et al. 2010; Tamura et al. 2013) using the gene-partitioned scheme mentioned above. For MrBayes, two parallel runs of 20 million generations were performed, with trees being sampled every 1000 generations using BEAGLE (high performance likelihood calculation library). Twenty percent of the generations were discarded as burn-in. For BEAST2, the analysis was run for 50 million generations and sampled every 10,000 generations. Ten percent of the sampled generations were discarded as burn-in. Using Tracer v. 1.6.0. (Rambaut and Drummond 2007), the effective sample size (**ESS**) was more than 200 for all parameters and the runs reached convergence, indicating that the burn-in for both BI phylogenies was adequate.

### Species delimitation

Species delimitation was used to elucidate whether the putative Angolan taxa identified in the phylogenetic tree constituted separate species. Outgroup taxa were removed, leaving only members of *Prosymna* for single locus species delimitation. The *16S* and *cyt-b* genes were chosen as they had the best representation. The following species delimitation analyses were used: Automatic Barcode Discovery (**ABGD**), Assemble Species by Automatic Partitioning (**ASAP**), Poisson Tree Processes (**PTP**), and Bayesian Poisson Tree Processes (**bPTP**).

Firstly, a *16S* and *cyt-b* alignment were prepared and uploaded onto the ABGD web interface (abgd web (mnhn.fr), web version 07 July 2022) and the ASAP Web Interface (ASAP web (mnhn.fr), web version 07 July 2022). For ABGD, the followings settings were used: standard p-distance metrics, minimum barcode gap width (1), intraspecific divergence minima (0.001) and maxima (0.1) (Puillandre et al. 2012). For ASAP, the Simple Distance (p-distances) substitution model was used (Puillandre et al. 2021).

Secondly, a *16S* and *cyt-b*ML tree were created in IQTREE, both using the GTR + I + G substitution model and the same settings implemented in the multi-locus phylogeny. The phylogenies were rendered as newick files and uploaded unrooted onto the bPTP web server (http://species.h-its.org/ptp/; Zhang et al. 2013) for PTP and bPTP analysis. The individual gene trees were then rendered, using Figtree v.1.4.2 (Rambaut 2014), and the results from the different single-locus species delimitation analyses were overlaid.

### Pairwise distance analysis

Pairwise distance analysis was implemented in MEGA X (Kumar et al. 2018) using the individual *16S* and *cyt-b* alignments, from the phylogenetic reconstruction. Sequences were trimmed to reduce missing data from the datasets and sequences that still had more than 10% data were removed from the alignments to ensure the most accurate p-distance values were attained. For the *16S* alignment, the hyper-variable region was retained. Sequences were grouped according to species and pairwise distance analysis was conducted using the following settings: uniform rates, pairwise deletion and 500 bootstrap replicates.

### Morphology

Morphological data was gathered from 39 *Prosymnaangolensis* sensu lato (Table 2). Snout-vent length (**SVL**, measured from the tip of the snout to the posterior end of the cloacal scale or vent opening) and tail length (**TL**, measured from the cloacal opening to the tip of the tail) were measured to the nearest 1 mm using a flexible ruler or a tape measure. We also expressed the TL as a percentage of the total length (SVL + TL). The following scale counts were recorded using a Nikon SMZ1270 binocular stereo microscope: number of middorsal scale rows (counted one head length behind head, at midbody, and one head length anterior to the cloacal scale), number of preoculars, number of postoculars, the temporal scale arrangement, number of supralabials and the number of supralabials entering orbit, number of infralabials and number of infralabials in contact with 1^st^ sublinguals, the presence of loreal, the number of ventral scales (Dowling 1951a), number of subcaudal scales (counted from anterior cloaca, excluding the terminal spine) and cloacal scale condition (divided or entire). Scale row reduction was also recorded (Dowling 1951b).

**Table 2.** Summary of morphological features and measurements for *Prosymnaangolensis* group. For abbreviations see Materials and methods. Notes: * including data on the Ebanga (Monard 1937) and Capelongo (Bogert 1940) specimens.

	*P.angolensis*	*P.angolensis*	*P.lisima* sp. nov.	*P.lisima* sp. nov.	*P.confusa* sp. nov.*
	Males	Females	Males	Females	Females
Sample size	6	21	7	3	3
SVL (mm)	160–248 (208.7 ± 29.8)	127–305 (224.7 ± 51.1)	138–198 (180.7 ± 20.5)	168–275 (214.3 ± 54.9)	231–240 (235.5± 6.4)
TL (mm)	22–30 (26.5 ± 2.7)	12–27 (29.9 ± 3.7)	17.0–28.9 (24.6 ± 3.7)	19.3–28.0 (22.6 ± 4.7)	23–29 (26.0 ± 4.2)
TL/total length ratio (%)	10.1–13.3 (11.40 ± 1.2)	6.5–9.4 (7.9 ± 0.9)	11.0–13.0 (12.0 ± 0.8)	8.8–10.9 (9.7 ± 1.1)	9.1–108 (9.9 ± 1.2)
Ventral scales	126–155 (138.1 ± 10.4)	134–163 (147.5 ± 8.3)	116–124 (120.2 ± 3.1)	117–129 (122.3 ± 6.1)	143–155 (149.7 ± 6.1)
Subcaudal scales	22–28 (25.4 ± 1.8)	16–25 (18.9 ± 2.5)	22–26 (23.7 ± 1.9)	18–24 (20.0 ± 3.5)	17–26 (21.0 ± 4.6)
Midbody scale rows	17-15-15 (rarely 19-15-15)		17-15-15		17-15-15
Cloacal scale	Entire		Entire		Entire
Preoculars	1		1 (rarely 2)		1
Postoculars	1 (rarely 0 or 2)		2		1
Temporals	1+2 (rarely 2+2, 2, 3)		1+2 (rarely 1+2+3)		1+5
Supralabials (contacting eye)	6 (3,4) [rarely 5 (2,3) or 7 (3,4)]		6 (3,4) [rarely 5 (2,3)]		5–6 (3, 4)
Infralabials (in contact with 1^st^ chin shield)	7 (3) [rarely 8 (3)]		7 (3)		7 (3)
Loreal	Present		Present		Present

For morphological comparison we preassigned material into three distinct groups based on the shared morphological and colouration differences observed by Broadley (1980) and our personal observations: 1) the nominotypical group which includes material (6 males; 21 females) from west/central Angola, northern Namibia, northern Botswana and north-eastern Zimbabwe (hereafter referred to as *P.angolensis* sensu stricto), 2) a group which includes material (7 males and 3 females) from eastern Angola, western Zambia and north-eastern Namibian (hereafter refer to as *Prosymna* ‘Eastern’), and a single female specimen from the coastal Angolan lowlands (PEM R24013) (hereafter refer to as *Prosymna* ‘Coastal’). We could not include the latter group in significant testing due to that we only had one confirmed sample.

To test if the ventral and subcaudal scale counts differs significantly between *Prosymna* ‘Eastern’ and *P.angolensis* sensu stricto as reported by Broadley (1980), we first corrected for size by dividing the ventral scale counts by the SVL and the subcaudal scale counts by the TL. The data was then separated by sex for further analyses. Due to our small sample size, we conducted a Shapiro-Wilk normality test and found that our data is not normally distributed and thus we proceed in conducting a non-parametric Wilcoxon test. We display the results using standard boxplots. All above-mentioned quantitative statistical comparisons were conducted using R v.4.1.0 (R Core Team 2021).

### Skull osteology

In order to identify diagnostic osteological characters and evaluate cranial variability within this group, we compared High Resolution X-ray Computed Tomography (**HRCT**) of newly collected material from Angola with *Prosymna* data provided by Heinicke et al. (2020), and two additional species (*P.janii* and P.cf.frontalis) not included in the previous study. We generated and analysed HRCT of three newly collected specimens from Angola (PEM R23512 [male], PEM R23510 [male] and PEM R24013 [female]), one specimen of *P.angolensis* (SAM ZR16574 [female]), one P.cf.frontalis (PEM R17997 [male]) and one *P.janii* (PEM R08679 [male]) at Stellenbosch University CT Scanner Facility using a General Electric Nanotom S system, using the settings specified in Appendix 1: Table A2. All specimens were regarded as adults. Three-dimensional segmentation models were generated for the articulated skull in Avizo Lite 2020.2 (Thermo Fisher Scientific 2020). To facilitate visualization, individual bone units for skulls and jaws were coloured following the same colour palette as Lobón-Rovira and Bauer (2021). All rendered files (*.ply) of premaxilla, palatine and maxilla, were analysed in Mesh Lab (2020.2) for colour processing and stacking of reconstructed in lateral, dorsal, ventral or medial views. Annotations were made in Adobe Illustrator CC 22.0.1 (Adobe Systems Incorporated 2017) following the anatomical terminology of Heinicke et al. (2020) and Broadley (1980). CT-scan raw data (.tiff files) have been deposited in MorphoSource (www.morphosource.org; Project ID 435270, Appendix 1: Table A2).

### Mapping

To enable the production of up-to-date occurrence maps for the *Prosymnaangolensis* group, data were sourced from published datasets (e.g., Broadley 1980, 1995; Marques et al. 2018) and museum databases. Online virtual museum platforms (http://www.inaturalist.org, http://vmus.adu.org.za, http://www.the-eis.com/atlas/; all accessed 31 July 2022) were also consulted and only a single record from the Namibian Atlas project was found that agreed with the diagnostic features identified by Broadley (1980) for *P.angolensis*. We gathered a total of 55 records that were assignable to *Prosymnaangolenis* sensu lato. The online GeoNames gazetteer (http://www.geonames.org/) or GEOLocate Web Application (https://www.geo-locate.org/web/WebGeoref.aspx) were used to georeference all historical data. Distribution data were mapped in QGIS v. 3.2 (http://qgis.org).

## Results

### Phylogenetic analyses

**Figure 2.**
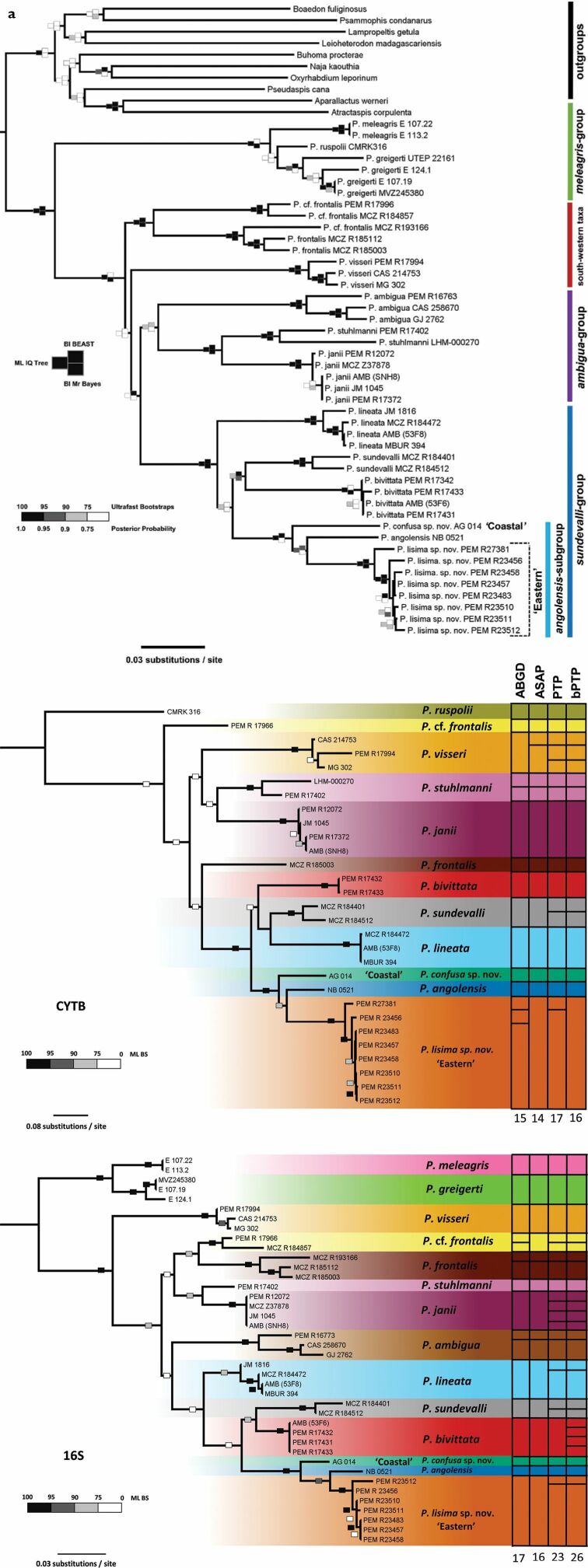
**a** Maximum likelihood (IQTREE) concatenated phylogeny with BI BEAST and BI MrBayes support overlain. Each of the species groups are demarcated by coloured vertical bars at the right margin **b** maximum likelihood (IQTREE) phylogenies (*16S* and *cyt-b*) with gene-specific single-locus species delimitation analyses results overlain. The bars to the right of the phylogeny represent the putative taxa assignments for each analysis and the values beneath the bars denote the total number of putative taxa for each analysis.

Maximum likelihood (bootstrap support [BS]) and both Bayesian Inference analyses (MrBayes posterior probability [MBPP]; BEAST posterior probability [BPP]) showed strong support for the monophyly of *Prosymna* (BS 100%, MBPP 1.0, BPP 1.0). (Fig. 2). The genus was characterised by four clades, identical to those found in Heinicke et al. (2020): *meleagris* group, south-western taxa, *ambigua* group, and *sundevalli* group. Whilst the inter-group structuring lacked support across the different algorithms, most of the relationships between sister species within the four groups were well supported.

The newly sequenced *P.angolensis* sensu lato material (hereafter ‘*angolensis* subgroup’) formed a monophyletic group, that was recovered within the *sundevalli* group by all three algorithms (BS 100%, MBPP 1.0, BPP 1.0). All three algorithms also retrieved identical topological sub-structuring within the *sundevalli* group, with strong support for the sister relationship between *P.lineata* and the rest of the species in the group. The sister relationship between *P.sundevalli* + *P.bivittata* and the *angolensis* subgroup was however not supported by any of the algorithms. Whilst all the phylogenies recovered the *angolensis* subgroup as monophyletic, they differed in their sub-structuring, with ML (BS 100%) and BEAST (BPP 1.0) recovering *Prosymna* ‘Coastal’ as sister to *Prosymna* ‘Eastern’ + *P.angolensis* sensu stricto and MrBayes (MBPP 1.0) recovering *P.angolensis* sensu stricto as sister to *Prosymna* ‘Coastal’ + *Prosymna* ‘Eastern’. All three algorithms recovered similar sub-structuring within the *Prosymna* ‘Eastern’ lineage with a specimen from Quembo River bridge camp (PEM R27381, Appendix 1: Table A1) being recovered as sister to the rest of the samples (BS 100%, MBPP 1.0, BPP 1.0).

The single locus ML phylogenies (Fig. 2b) produced similar topological structuring to the concatenated dataset, albeit with markedly lower support. Where the topologies do differ, the nodes lack support. Although inter-group support is low, the intra-group support is high and is similar to that observed in multi-locus phylogeny (Fig. 2a). Similar to the multi-locus phylogeny (Fig. 2a), the novel Angolan specimens were recovered as a monophyletic clade with both *16S* (BS 100%) and *cyt-b* (BS 100%) recovering *Prosymna* ‘Coastal’ as sister to *Prosymna* ‘Eastern’+ *P.angolensis* sensu stricto.

The species delimitation analyses employed across both the *16S* and *cyt-b* phylogenies recovered a substantial amount of putative taxa, with vastly different estimates between the different analyses. Across both phylogenies, ABGD and ASAP were more conservative and PTP and bPTP were more liberal with the number of putative taxa. Whilst the differential sampling afforded to the different gene phylogenies resulted in differing numbers of putative taxa, it must be noted that all four species delimitation methods recognised *Prosymna* ‘Eastern’, *Prosymna* ‘Coastal’ and *P.angolensis* sensu stricto as independent species when using both the *16S* and *cyt-b* marker. While not the focus of this study, notable cryptic speciation was also recovered in *P.frontalis*, *P.ambigua*, and *P.stuhlmanni* when using either the *16S* or *cyt-b* marker.

The average *16S* pairwise divergence separating species within the genus was 8.11% (± 0.23% s.e. – standard error) when the *angolensis* subgroup is considered a single species (Table 3). While the average pairwise divergences separating the three lineages of the *angolensis* subgroup from the rest of the genus varies from 6.99% (± 0.52% s.e.) to 7.99% (± 0.67% s.e.). Although the *angolensis* subgroup is well delineated from the rest of the genus, when these lineages are compared to one another, the *angolensis* subgroup was characterised by relatively low pairwise divergences. *Prosymnaangolensis* sensu stricto was separated from *Prosymna* ‘Eastern’ material by an average pairwise distance of 2.92% (± 0.75% s.e.) and from the *Prosymna* ‘Coastal’ sample by 3.75% (± 0.89% s.e.), while the *Prosymna* ‘Eastern’ and *Prosymna* ‘Coastal’ material were separated by 4.76% (± 0.99% s.e.).

For the *cyt-b* gene, the average pairwise divergence separating species of the genus was 20.60% (± 0.42% s.e.) (Table 3). The average pairwise divergences separating the three lineages of the *angolensis* subgroup from the rest of *Prosymna* varies from 18.39% (± 1.11% s.e.) to 19.08% (± 0.95% s.e.). Like the *16S* gene, the *angolensis* subgroup was characterised by lower pairwise divergences when these three lineages were compared to each other, albeit relatively higher than what was found when using the *16S* gene. *Prosymnaangolensis* sensu stricto was separated from *Prosymna* ‘Eastern’ material by a pairwise distance of 13.90% (± 1.31% s.e.) and from *Prosymna* ‘Coastal’ material by a pairwise distance of 13.25% (± 1.40% s.e.), while eastern *Prosymna* ‘Coastal’ material is separated from the *Prosymna* ‘Eastern’ specimen by 15.55% (± 1.47% s.e.).

**Table 3.** Sequence divergence (uncorrected pairwise distance values) for *16S* and *cyt-b* separating the species of *Prosymna*. Numbers in the diagonal (in bold) denote intraspecific divergences, numbers below the diagonal denote interspecific divergences and numbers above the diagonal denote the standard error of the interspecific divergences. NA–Not Available.

*16S*														
		1	2	3	4	5	6	7	8	9	10	11	12	13
1	*P.ambigua*	**3.02**	1.28	1.19	1.23	1.07	1.28	1.15	1.03	1.36	1.31	1.13	1.22	1.19
2	*P.angolensis*	8.58	**NA**	1.19	0.89	1.08	1.37	1.21	1.21	0.75	1.39	1.23	1.16	1.30
3	*P.bivittata*	7.28	6.26	**0**	1.00	1.12	1.39	1.35	1.09	1.28	1.41	1.23	0.95	1.31
4	*P.confusa* sp. nov. ‘Coastal’	8.34	3.75	5.07	**NA**	1.11	1.28	1.29	1.15	0.99	1.30	1.25	1.01	1.32
5	*P.frontalis*	8.11	7.71	8.01	8.14	**5.28**	1.22	1.01	0.99	1.15	1.23	0.93	1.08	1.13
6	*P.greigerti*	9.55	9.96	10.44	8.82	9.84	**1.55**	1.28	1.30	1.38	0.64	1.34	1.36	1.28
7	*P.janii*	7.51	7.32	8.63	7.96	6.75	8.35	**0**	1.02	1.30	1.24	0.93	1.29	1.23
8	*P.lineata*	6.50	6.73	5.87	6.39	6.93	9.44	5.78	**0.69**	1.22	1.27	0.92	1.07	1.20
9	*P.lisima* sp. nov. ‘Eastern’	10.06	2.92	7.53	4.76	8.56	10.85	8.53	7.36	**0.63**	1.40	1.30	1.25	1.27
10	*P.meleagris*	9.62	10.11	10.31	9.16	10.26	2.82	8.39	9.47	11.03	**2.52**	1.28	1.39	1.32
11	*P.stuhlmanni*	7.58	7.76	7.51	7.52	6.16	9.24	3.96	5.07	8.37	9.31	**NA**	1.20	1.25
12	*P.sundevalli*	8.56	7.63	4.97	5.63	7.85	11.11	8.92	6.32	8.16	11.68	8.04	**1.54**	1.28
13	*P.visseri*	7.88	8.38	8.01	8.36	8.01	9.07	7.40	7.90	7.76	9.49	7.77	8.64	**0.74**
***cyt-b***														
		**1**	**2**	**3**	**4**	**5**	**6**	**7**	**8**	**9**	**10**	**11**		
1	*P.angolensis*	**NA**	1.43	1.40	1.38	1.58	1.56	1.31	1.67	1.70	1.39	1.64		
2	*P.bivittata*	15.76	**0.33**	1.41	1.32	1.60	1.55	1.57	1.70	1.72	1.40	1.64		
3	*P.confusa* sp. nov. ‘Coastal’	13.25	16.35	**NA**	1.38	1.62	1.60	1.47	1.70	1.72	1.32	1.74		
4	*P.frontalis*	19.95	19.72	19.28	**17.97**	1.44	1.45	1.44	1.49	1.35	1.34	1.47		
5	*P.janii*	19.64	21.50	19.91	20.17	**1.08**	1.59	1.57	1.72	1.62	1.51	1.67		
6	*P.lineata*	18.92	17.84	18.36	20.18	20.16	**0.22**	1.55	1.82	1.73	1.38	1.66		
7	*P.lisima* sp. nov. ‘Eastern’	13.90	18.73	15.55	21.38	19.75	17.67	**1.58**	1.71	1.69	1.39	1.58		
8	*P.ruspolii*	23.86	24.05	23.75	23.45	24.40	25.26	23.81	**NA**	1.80	1.67	1.69		
9	*P.stuhlmanni*	20.75	20.92	20.83	18.79	17.97	20.52	20.68	23.47	**NA**	1.45	1.65		
10	*P.sundevalli*	15.99	16.08	14.63	19.51	18.97	15.39	17.61	23.70	17.52	**6.49**	1.59		
11	*P.visseri*	21.87	22.29	22.38	21.27	20.33	22.57	21.73	25.04	19.56	20.49	**3.09**		

### Morphology

**Figure 3.**
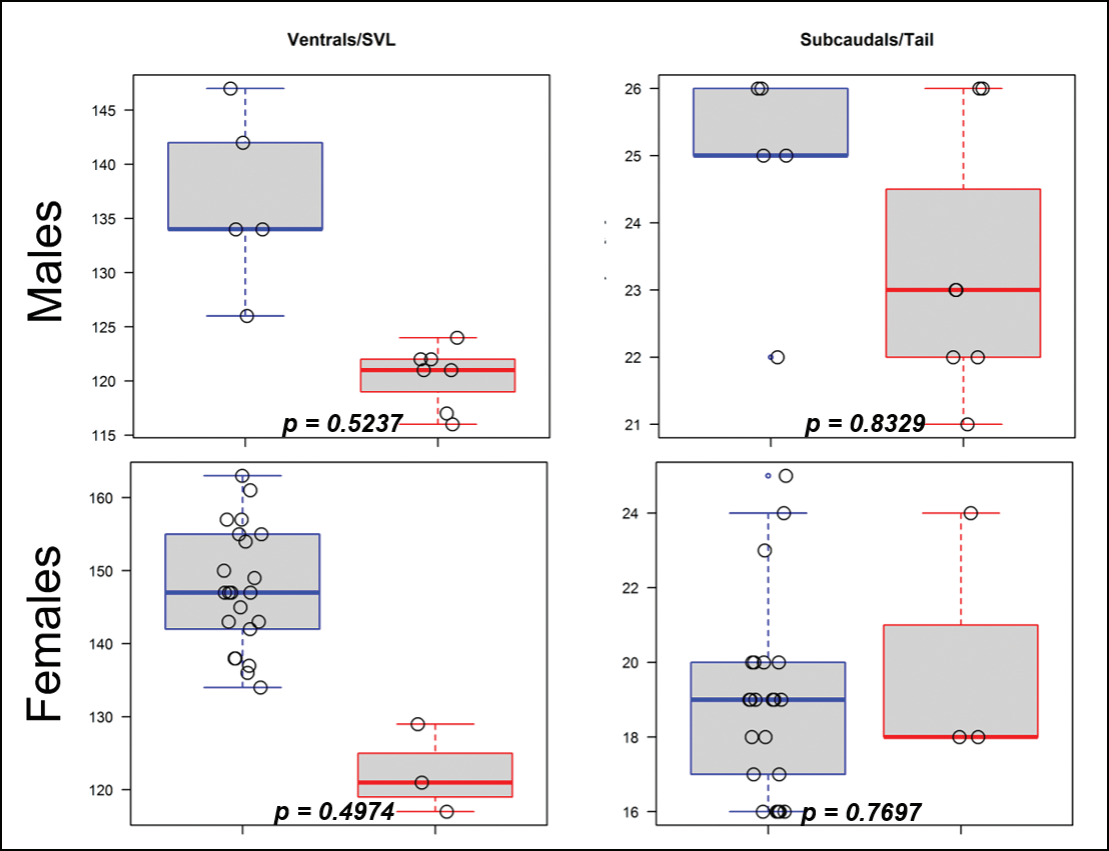
Summary boxplots (top whisker–maximum value; lower whisker–minimum value; dark horizontal line–median; box–1^st^ and 3^rd^ quartiles, open circles–data points) comparing ventral and subcaudal scales corrected for size among the species of *Prosymna* separated by sex: *Prosymnaangolensis* (blue) and *Prosymnalisima* sp. nov. (red); *p*-value of non-parametric Wilcoxon test is indicated at bottom of each boxplot. *Prosymnaconfusa* sp. nov. is not included due to small sample size.

There was a degree of overlap in most morphological features (measurements and scale counts) for all the material examined (Table 2). Although *Prosymna* ‘Eastern’ has lower number of ventral scales (116–129 [average 121] compared to *Prosymnaangolensis* sensu stricto (126–163 [average 145]), the results of the non-parametric Wilcoxon test showed that there are no significant differences in the ventral and subcaudal scale counts when corrected for size for both sexes (Fig. 3). The only other consistent scalation differences between the *P.angolensis* sensu stricto and *Prosymna* ‘Eastern’ material, was the number of postoculars: predominantly has one postocular (only 4 of 31 had two postoculars) vs. always have two postoculars (*n* = 10), respectively. Due to small sample size, the morphology of the single specimen of *Prosymna* ‘Coastal’ could not be statistically compared with the other two lineages. It exhibited similar scalation to *P.angolensis* sensu stricto (i. e., presence of a higher ventral scales count and presence of a single postocular) (Table 2), but differ in dorsal colouration (see below).

### Colouration

**Figure 4.**
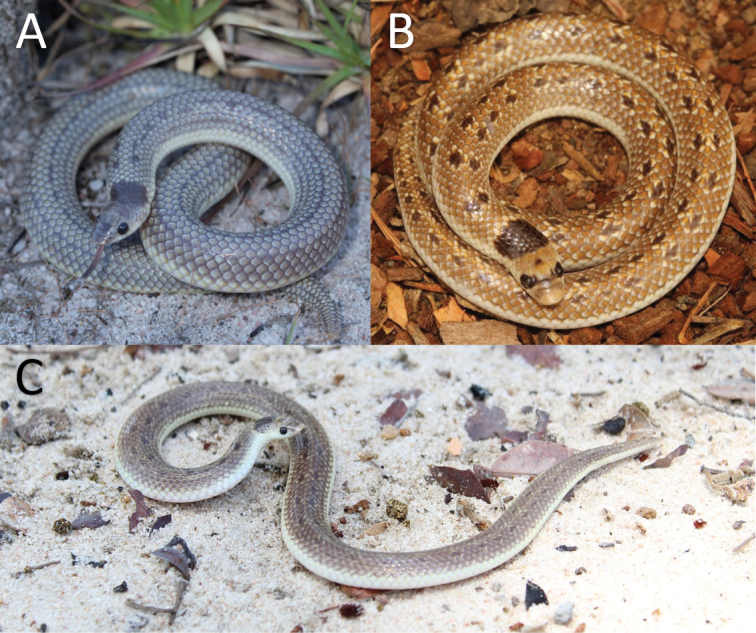
Variation in dorsal colouration of nominotypical *Prosymnaangolensis***A** Tundavala, Huíla Province, Angola (Photo: Justin R. Nicolau) **B** Grootfontein, Namibia (Photo: Francois Theart) **C** Bicuar National Park (CHL 0521), Huíla Province, Angola (Photo: Ninda L. Baptista).

**Figure 5.**
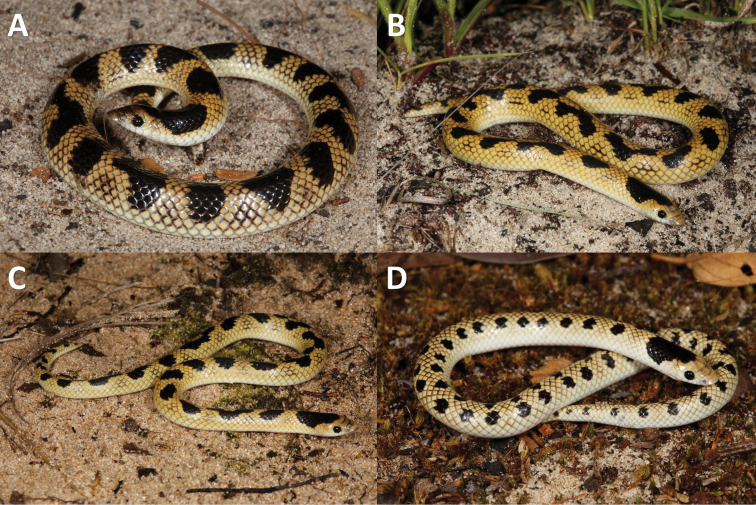
Photos of live *P.lisima* sp. nov. from eastern Angola **A**PEM R23457 from Quembo River Source, Moxico Province, Angola **B**PEM R23483 from Cuando River Source, Moxico Province, Angola **C**PEM R23512 from Cuito Source Lake, Moxico Province, Angola **D**PEM R27381 from Quembo River bridge camp, Moxico Province, Angola.

**Figure 6.**
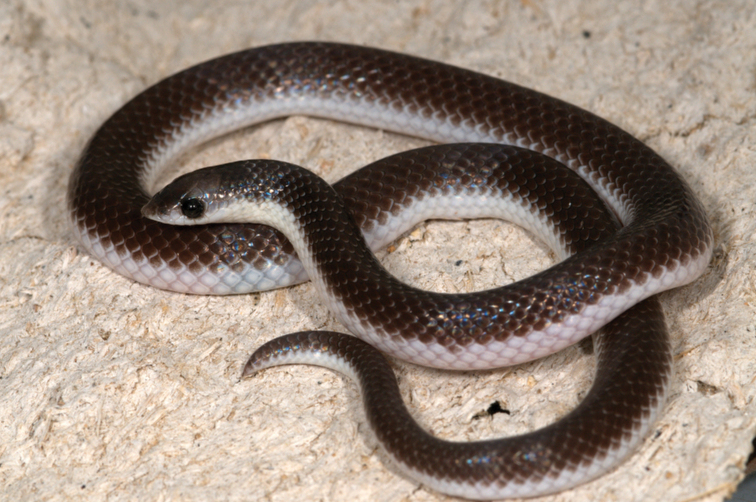
Live *P.confusa* sp. nov. (PEM R24013) from 20 km west of Lola on the road northwest to Camacuio and on the edge of Bentiaba River, Namibe Province, Angola (Photo: Bill Branch).

The colouration of *P.angolensis* sensu stricto (24 out of 28 examined) varied from pale grey to yellow-brown with a large black bar behind the head and a series of smaller paired black spots along the back, similar to *P.sundevalli*. In some cases (2 out of 28 examined), these black spots are very faint and disappear anteriorly or form continuous faint paravertebral stripes (2 out of 28 examined), similar to *P.lineata* (Fig. 4). On the other hand, all material from *Prosymna* ‘Eastern’ (*n* = 10) exhibited a golden yellow dorsum colouration with a large black bar behind the head, followed by fused irregular black blotches along the back continuing onto the tail (Fig. 5). The only subadult male collected had smaller paired dorsal black spots, similar to the main population but more defined (Fig. 5D). Although we could not statistically compare the morphology of the *Prosymna* ‘Coastal’ specimen, it has uniform dark grey dorsum with a very faint black bar behind the head (Fig. 6).

### Skull osteology (Figs 7–9)

The osteological analysis has shown that material within the *angolensis* subgroup presents the same common features shared across *Prosymna*: a compact and rigid skull, anterior reduction of the maxilla, enlargement of the posterior maxillary teeth and reduced palatine, and a unique tooth loci formula with seven reduced tooth loci and four or five posterior lancet-shaped and enlarged tooth loci (see Heinicke et al. 2020). Some specimens in the *angolensis* subgroup had more than four frontal foramina present, such as in *P.angolensis* sensu stricto and *Prosymna* ’Eastern’, vs. one or two in *Prosymna* ‘Coastal’.

**Figure 7.**
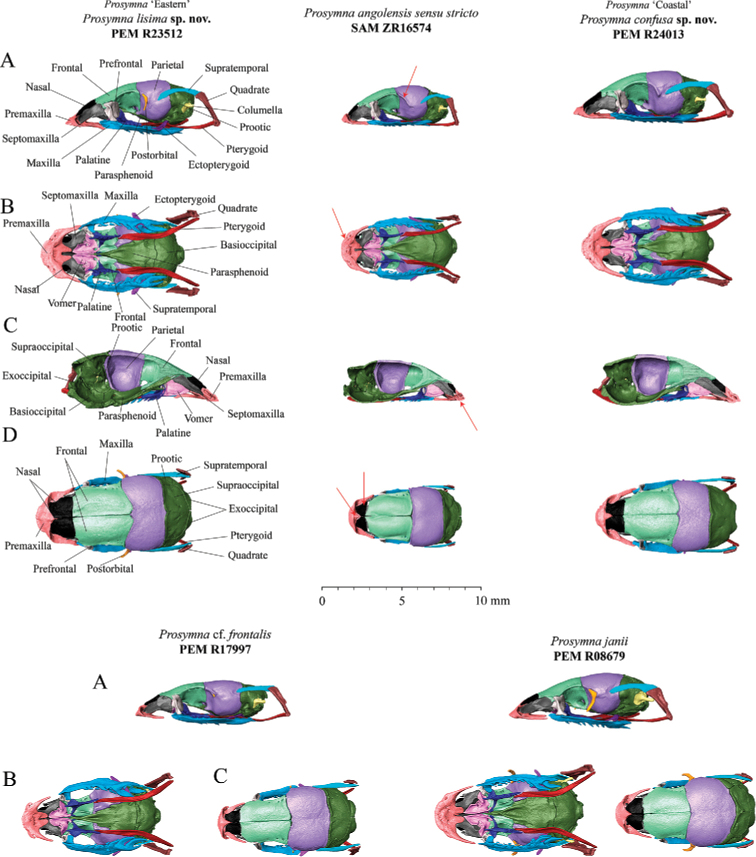
**A** Lateral **B** ventral **C** medial **D** dorsal views of skulls of *Prosymnaangolensis* subgroup. Red arrows depict variation characters between the *P.lisima* sp. nov., *P.angolensis*, *P.confusa* sp. nov., P.cf.frontalis, and *P.janii*.

The *angolensis* subgroup shares some cranial features with the rest of the *sundevalli* group, such as an elongated maxillary process, with the premaxilla being in contact with the maxilla and the lack of a postorbital bone (except *Prosymna* ‘Eastern’, see below). It differs from the *sundevalli* group by the absence of partial or total fusion between the braincase and the parietal bone and the absence of lateral tubercles in the premaxilla in *angolensis* subgroup (vs. present in *sundevalli* group).

**Figure 8.**
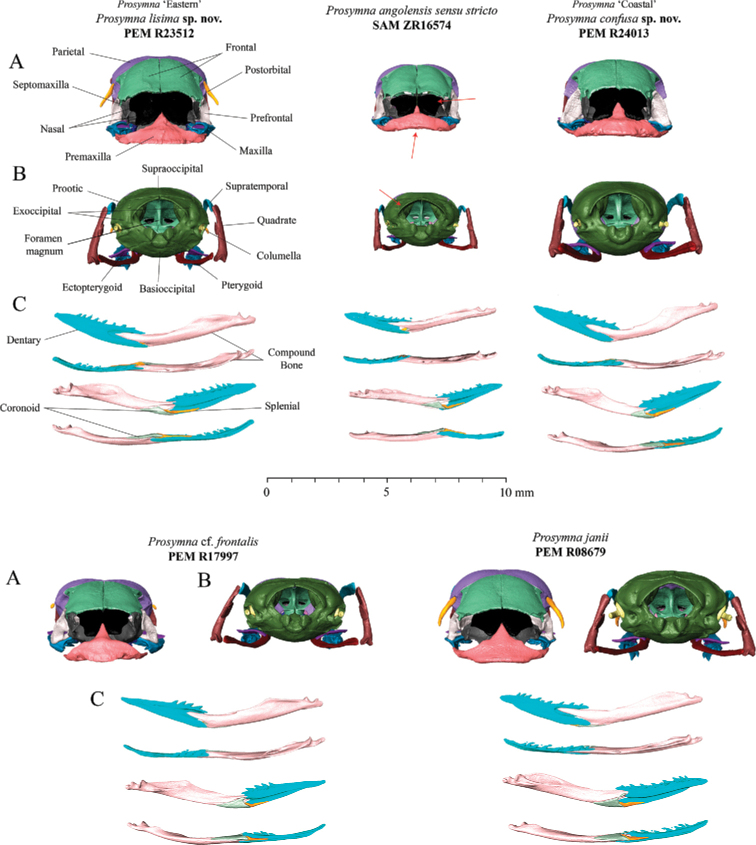
**A** Frontal and **B** posterior views of skulls, and **C** lateral, dorsal, medial and ventral view of jaw of *Prosymnaangolensis* subgroup. Red arrows depict variation in characters between the *P.lisima* sp. nov., *P.angolensis*, *P.confusa* sp. nov., P.cf.frontalis, and *P.janii*.

The *Prosymna* ‘Eastern’ material is unique in possessing a well-developed postorbital bone, which is shared with P.cf.frontalis (although much more reduced) and the *ambigua* group (well developed, including here corroborated for *P.janii*). Two of the *angolensis* subgroup lineages (*P.angolensis* sensu stricto and *Prosymna* ‘Eastern’) present an unfused braincase (only known to be present in *ambigua* group, here corroborated for *P.janii*); however, the *Prosymna* ‘Coastal’ specimen presents a fused braincase.

**Figure 9.**
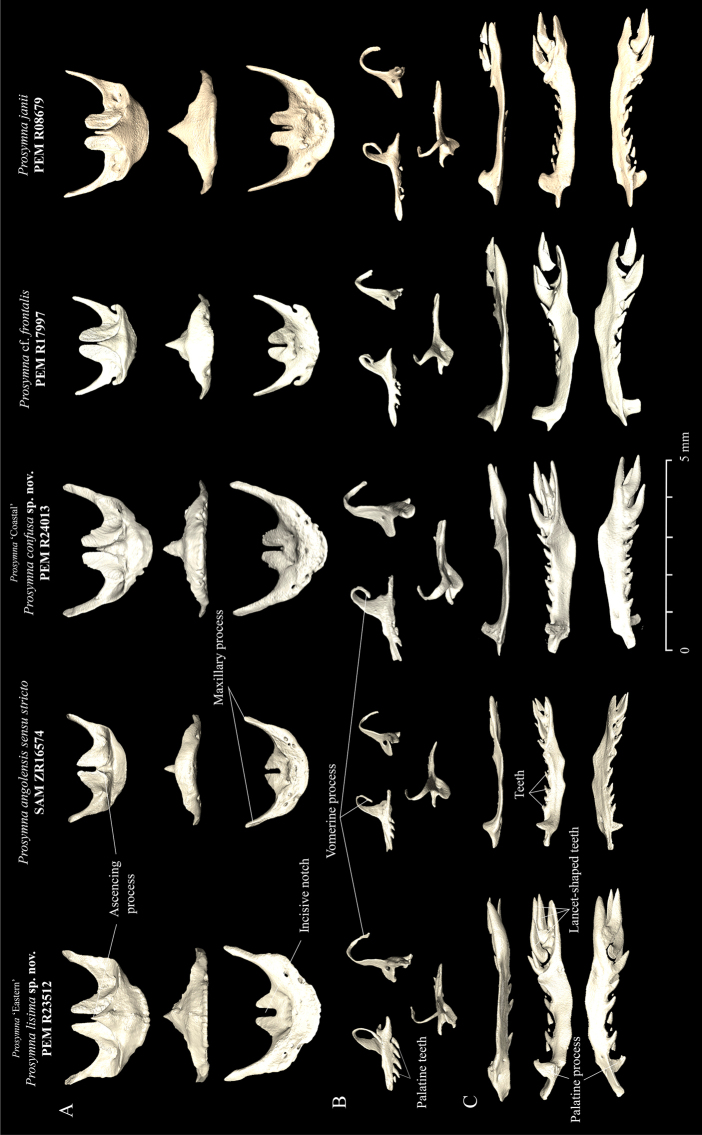
**A** Dorsal, frontal and ventral view of premaxillae **B** medial, posterior and dorsal views of palatine; and **C** lateral, dorsal, and ventral view of maxilla in *P.lisima* sp. nov., *P.angolensis*, *P.confusa* sp. nov., P.cf.frontalis and *P.janii* (from left to right).

Finally, the osteological comparison demonstrated the presence of unique diagnostic features between the three lineages from the *angolensis* subgroup, i.e., presence/absence of postorbital bone, fused/unfused braincases, and the number of palatine teeth and frontal foramina, which are addressed in more detail below.

### Systematics

Based on the genetic, morphological, and colouration differences discussed above, we recognise all three lineages within the *angolensis* subgroup as independently evolving lineages and describe two (*Prosymna* ‘Eastern’ and *Prosymna* ‘Coastal’) of them as new species. We follow the general lineage-based species concept (de Queiroz 1998).

#### Reptilia: Squamata: Prosymnidae

##### Prosymna angolensis

Taxon classificationAnimaliaSquamataProsymnidae

Boulenger, 1915

47DE6855-35F0-518C-8E1A-C4092C68D514

**Figure 10.**
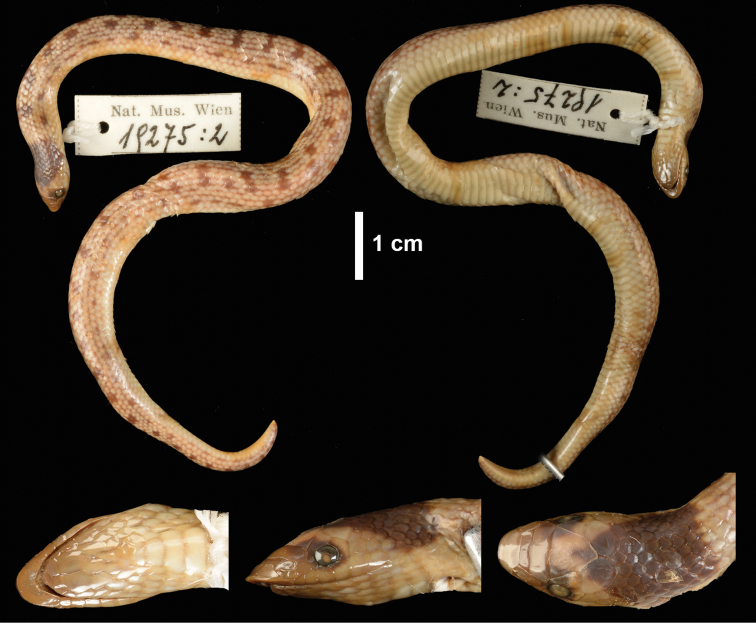
Neotype of *Prosymnaangolensis* (NMW 19275:2) from Caconda, Huíla Province, Angola (Photos: Alice Schumacher, Natural History Museum Vienna).

Figs 4 , 7 , 8 , 9 , 10 Common names: Angolan Shovel-snout snake (English); Cobra-de-focinho-de-pá-de-Angola (Portuguese).

###### Chresonymy.^1^

*Prosymnafrontalis*: Bocage 1873: 218, 1882: 288, 1895: 98; Boulenger 1894: 248, 1896: 641.

*Prosymnaambigua*: Monard 1931: 104, 1937: 123; Mertens 1937: 13.

*Prosymnaambiguaambigua*: Mertens 1938: 439; Loveridge 1958: 151.

*Prosymnaangolensis*: Boulenger 1915: 208; Chabanaud 1916: 439; Monard 1937: 114, 122; Bogert 1940: 59; Mertens 1955: 94, 1971: 86; Hellmich 1957: 66; Loveridge 1958: 149; FitzSimons 1962: 161, 1966: 53, 1970: 104; Isemonger 1968: 129; Broadley 1980: 512, 1990: 227, 1995: 48; Auerbach 1987: 178; Branch 1998: 84, 2018: 64; Broadley et al. 2003: 187 (in part); Marais 2004: 236; Broadley and Blaylock 2013: 219; Herrmann and Branch 2013: 8; Wallach et al. 2014: 568; Baptista et al. 2019: 118; Chippaux and Jackson 2019: 283, Ceríaco et al. 2021a: 16 (in part).

When Boulenger (1915) described *P.angolensis*, he did not designate a precise type locality nor a type specimen for that matter. Later, Loveridge (1958) proposed designating Huíla as the type locality, but it was Broadley (1980) that finally restricted the type locality to Caconda by designating a lectotype of the material he examined on his visit to Museu Bocage, Lisbon (MBL), Portugal in 1968. The reasons for this change in the proposed type locality, were that the Huíla specimen was unaccounted for, as well as the Caconda material was in the best overall condition to represent the species. He initially designated MBL 1606b as the lectotype, but with the destruction of the MBL collection, he designated one of the remaining Caconda specimens in Naturhistorischen Museums in Wien (NMW 19275b) as the replacement neotype (see Gemel et al. 2019).

###### Material examined.

***Neotype* (Fig. 10).** NHMW 19275:2, collected from Caconda (approx. -13.73537, 15.06720, 1662 m a.s.l.), Huíla Province, Angola. Neotype designated by Donald Broadley (1980). ***Additional material*.**MBL 1609, Angola (no precise locality), Angola; MBL 1605a, Bibala, Angola; MBL 1605b, Bibala, Angola; CHL 0521, Bicuar NP, Angola; NMW 19275:1, Caconda, Angola; NMW 19275:2, Caconda, Angola; MBL 1606a, Caconda, Angola; MBL 1606b (original lectotype), Caconda, Angola; MBL 1606c, Caconda, Angola; MBL 1608, Caconda, Angola; USBN 20035, Luanda, Angola; CAS 84181, Luanda, 3 mile S of airport, Angola; MBL 1607, Maconjo = Maconge, Angola; MCZ 32468, Missão do Dondi Bela Vista, Angola; MBL 1604, interior of Mossamedes, Angola; UM 20178, Goeverega, Botswana; UM 21271, 15 km WSW of Katima Mulilo, Namibia; SMF 46614, Karakuwisa, Kavango, Namibia; TM 55043, Katima Mulilo, Namibia; UM 24204, Katima Mulilo, Namibia; SAM ZR16574, Namutoni, Namibia; NMZB 9532, NE of Waterberg, Namibia; NMZB 13953, Inyokene, Nyamandhlovo, Zimbabwe; NMZB 13787, Malinbdi Siding, Hwange, Zimbabwe; NMZB 13788, Malinbdi Siding, Hwange, Zimbabwe.

###### General description.

See Table 2 for summarised meristic data. Dorsal scales smooth, arranged in 17-15-15 (rarely 19-15-15) rows at midbody, scale row reduction takes place between ventral scales 16–20 (males) and 14–49 (females); one (sometimes two or three on supracaudal scales) apical pits; 126–163 (126–155 males, 134–163 females) smooth ventral scales; 16–28 (22–28 males, 16–25 females) paired subcaudal scales; rostral is acutely angular horizontally; internasal is single and bandlike; 1 preocular; 1 (rarely 0 or 2) postocular; temporals mostly 1+2 (rarely 2+2, 2+3); mostly 6 supralabials, with 3^rd^ and 4^th^ entering the orbit (rarely 5 (2, 3) or 7 (3, 4)); 7 infralabials, with first 3 in contact with the chin shield (rarely 8 (3)), cloacal scale entire.

***Skull osteology and teeth* (Figs 7**–**9).** Based on the examination of a single female specimen (SAM ZR16574, Namutoni, Namibia), *P.angolensis* presents a compact and rigid skull, which is common among *Prosymna* species. It has an unfused braincase and nasal bones. Parietals are fused and the fronto-lateral portion presents a sharp edge that forms the edge of the orbital rim. Postorbital bone is absent. Premaxilla has a reduced ascending nasal process with a small groove between the ascending process and frontal portion of the bone. Premaxilla lies between the ventral laminae of nasals with a high profile of the anterior portion which curves shapely to finish on a convex profile. Maxilla and premaxilla are in contact. Nasal bones are reduced and display a wing-shape with a narrower anterior portion. Septomaxilla is a well-developed bone, in broad contact with the premaxilla, frontal, vomer, prefrontal and frontal bones. Vomer well developed with perforated dorsolateral portion of the bone. Maxilla reduced anteriorly with an elongated pick-shaped palatine process, with seven or eight laterally reduced curved tooth loci, followed by four enlarged and lancet-shaped tooth loci. Palatine with three reduced teeth and an enlarged dorsal and curved vomerine process that reinforces the internal portion of the orbit. Pterygoid is a thin elongated bone. Supratemporal is an enlarged bone in broad contact with the quadrate and participates in the lateral movement of the lower jaw. The lower jaw consists of compound, splenial, coronoid, and dentary bones. Coronoid and splenial bones are reduced, almost vestigial. Dentary with eight or nine small sharp tooth loci, with first third clear of any teeth.

***Colouration in life* (Fig. 4).** The head is yellowish-brown with variable darker black markings that can be absent. Most commonly there is an anterior black band across the frontal, followed by a pair of black blotches around the orbits, supraoculars and parietals. A distinct black nuchal spot or collar is often present. The dorsal colouration varies from having mostly small paired black longitudinal vertebral spots (similar to *P.sundevalli*) to a speckled pattern (similar to *P.lineata*) on a pale yellowish-brown to grey ground colour. Ventrum and outermost two or three dorsal scale rows yellowish white.

***Hemipenis*.** Short hemipenis with a length that reaches the 9–10^th^ ventral scales (Broadley 1980).

***Size*.** Males vary from 160–248 (208.7 ± 29.8) mm SVL and 22–30 (26.5 ± 2.7) mm TL, with the largest male measuring 248+28 = 276 mm (NMZB 9532, NE of Waterberg, Namibia). Females vary from 127–305 (224.7 ± 51.1) mm SVL and 12–27 (19.9 ± 3.7) mm TL, with the largest female measuring 305+22 = 327 mm (SMF 32541, Cubal, Angola). Bocage (1895) mentioned an unsexed individual (probably a female) that measured 331+29 = 360 mm, but this specimen was unaccounted for in MBL.

###### Natural history.

This is a semi-fossorial species that feeds exclusively on reptile eggs, using its blade-like rear maxillary teeth to puncture the eggs, similar to other *Prosymna* species.

###### Distribution and habitat.

Currently the species is known to occur in three main geographic clusters: west-central Angola, north-central Namibia and isolated records from the Zambezi Region in north-eastern Namibia, northern Botswana and north-western Zimbabwe (Fig. 2). However, it is possible that this distribution might be more continuous, given this is a rarely observed species that mostly emerges to the surface only after good rains (Heinicke et al. 2020). The records from Luanda (USBN 20035 and CAS 84181) and northern Angola (IICT/R 14-1957) require verification. This species is associated with savanna with an annual rainfall of 500–1200 mm (Broadley 1980). In southwestern Angola it has been found in miombo woodland in sandy soils (Baptista et al. 2019). In the eastern Zambezi Region and northern Botswana, the species is associated with drier savanna in deep Kalahari sands (Broadley 1980).

###### Localities.

**Angola**: Bela-Vista (Missão do Dondi), -12.36667, 16.20000 (Hellmich 1957: 66); interior of Benguela (Bocage 1895: 98); Bibala, -14.76667, 13.36667 (Bocage 1873: 218); Bicuar National Park, Woodland trapline 1, -15.09441, 14.83831 (Baptista et al. 2019: 118); Caconda, -13.73537, 15.06720 (Bocage 1895: 151); Cubal, -13.03333, 14.73333 (Mertens 1938: 439); Ebanga, -12.73333, 14.73333 (Monard 1937: 123); Huíla, -15.08333, 13.55000 (Bocage 1895: 98); Luanda and ‘Luanda, 3 mi S of airport’, -8.83333, 13.26667 (Broadley 1980: 515); Maconjo, -15.016667, 13.2000 (Bocage 1895: 98); interior of Mossamedes (Bocage 1873: 218); Quibula, -12.28333, 14.68333 (Bocage 1895: 98); Posto do Milando (-8.81667, 17.56667) (Ceríaco et al. 2021a: 16). Quindumbo, -12.46667, 14.93333 (Bocage 1895: 98); Quissange, -12.43333, 14.05000 (Bocage 1895: 98); Tundavala, -14.82018, 13.404217 (Justin Nicolau photo). **Botswana**: Joverega (Geoverega), -19.13333, 24.25 (Broadley 1980: 515). **Namibia**: Grootfontein, -19.55012, 18.10965 (Francois Theart photo); Karakuwisa, -18.933333, 19.733333 (Mertens 1955: 94); Katima Mulilo, -17.5, 24.266667 (Broadley 1980: 515); Namutoni, -18.807624, 16.940288 (FitzSimons 1962: 161); 15 km WSW of Katima Mulilo, -17.61448, 24.205932 (Broadley 1980: 515); Otjozondjupa Region, -19.08800, 18.83300 (http://www.the-eis.com/atlas/?q=details/snake-record&occurrence_id=654386). **Zimbabwe**: Malindi Siding, Hwange, -18.74885, 27.01852 (Broadley 1995: 48); Inyokene, Nyamandlovu, -19.93333, 28.06667 (Broadley 1995: 48).

##### Prosymna lisima sp. nov.

Taxon classificationAnimaliaSquamataProsymnidae

C426FC38-04F0-5675-ADAC-BDAF58995DB5

https://zoobank.org/70A338BA-ED44-440B-AE3B-863E84B3AFBE

**Figure 11.**
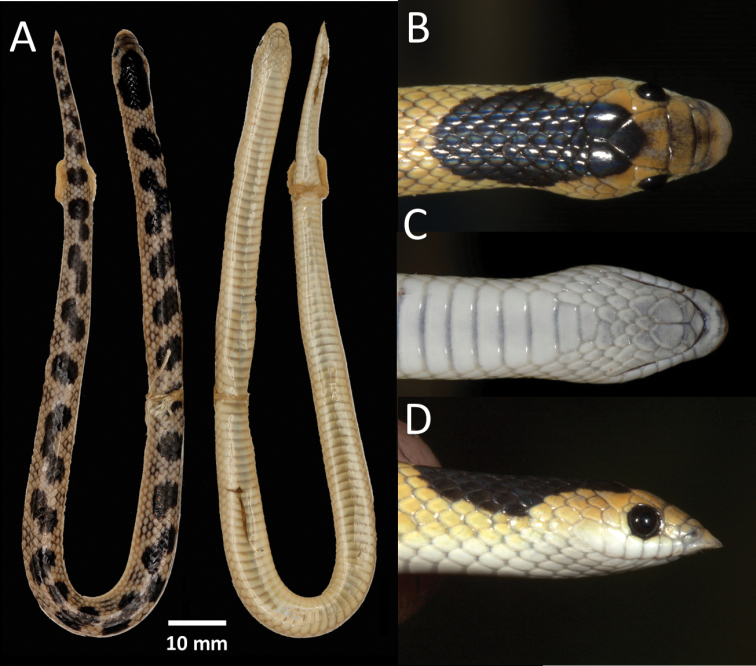
Holotype of *Prosymnalisima* sp. nov. (PEM R23512) from Cuito River source, Moxico Province, Angola **A** dorsal and ventral full body **B** dorsal head **C** ventral head **D** lateral head.

**Figure 12.**
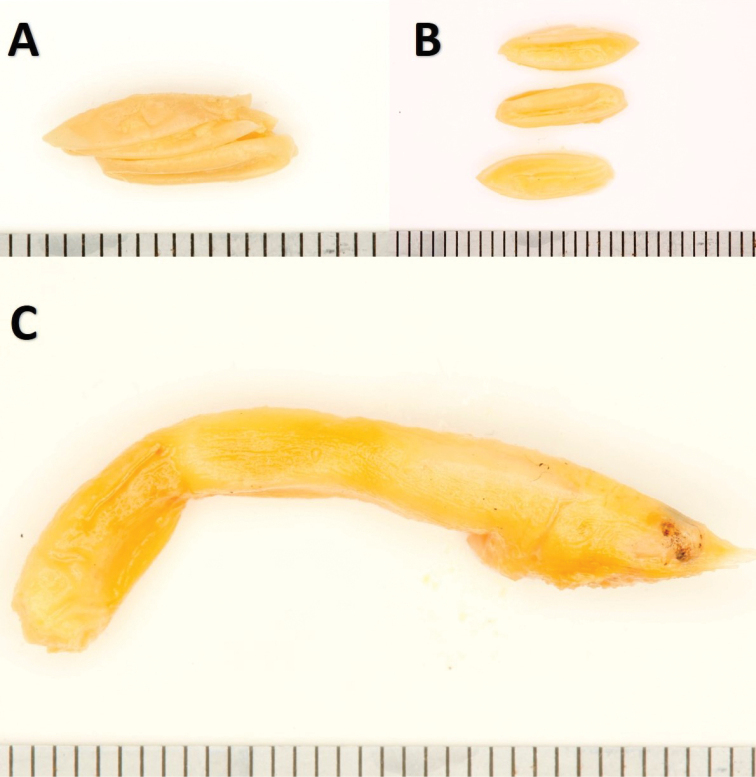
Stomach and gut contents of *Prosymnalisima* sp. nov. (PEM R23456) consisting of lizard egg shells. Scale bar: 1 mm.

Figs 5 , 7 , 8 , 9 , 11 , 12 Common names: Kalahari Shovel-snout snake (English); Cobra-de-focinho-de-pá-do-kalahari (Portuguese).

###### Chresonymy.

*Prosymnaangolensis*: Broadley 1971: 82, 1980: 512 (in part); Broadley et al. 2003: 187 (in part); Pietersen et al. 2021: 97, fig.; Conradie et al. 2021: 265.

###### Material examined.

***Holotype*** (male). PEM R23512, collected from Cuito River source lake (-12.68866, 18.36025, 1426 m a.s.l.), Moxico Province, Angola by Werner Conradie and James Harvey on 26 November 2016. ***Paratypes*** (five males). PEM R27381, collected from Quembo River lower bridge (-13.526579, 19.278096, 1248 m a.s.l.), Moxico Province, Angola by Werner Conradie, Chad Keates and Timóteo Júlio on 27 November 2019; PEM R23457–8, collected from Quembo River source (-13.13586, 19.04709, 1368 m a.s.l.), Moxico Province, Angola by Werner Conradie on 3 November 2016; PEM R23483, Cuando River source (-13.00164, 19.1296, 1372 m a.s.l.) Moxico Province, Angola by Werner Conradie and James Harvey on 17 November 2016; PEM R23510, collected from Cuito River source lake (-12.68866, 18.36025, 1426 m a.s.l.), Moxico Province, Angola by Werner Conradie and James Harvey on 26 November 2016. ***Paratypes*** (two females). PEM R23456, collected from Quembo River source (-13.13586, 19.04709, 1368 m a.s.l.), Moxico Province, Angola by Werner Conradie on 3 November 2016; PEM R23511, Cuito River source lake (-12.68866, 18.36025, 1426 m a.s.l.), Moxico Province, Angola by Werner Conradie and James Harvey on 26 November 2016.

###### Additional material assigned to the new species.

NMZB-UM 10096, Kalabo (approx. -14.99391, 22.67795), Zambia; NMZB-UM 21272, 15 km WSW of Katima Mulilo (approx. -17.61448, 24.20593), Namibia. A specimen from Kuvangu [= Vila-da-Ponte], -14.46667, 16.3000 (Monard 1937: 123) has two postoculars and the characteristic confluent blotched dorsal pattern and might belong to this new species, but this needs verification and is thus tentatively referred to the new species.

###### Diagnosis.

The new species differs from other *Prosymna* in the following characters: rostral sharply depressed and angular (vs. rounded in *P.visseri*); presences of a single band-like internasals (vs. paired internasals in *P.somalica*, *P.bivittata*, *P.sudevalli*, *P.lineata*); dorsal scales smooth (keeled in *P.janii*); midbody scale rows 15–17 (vs. 19–21 in *P.pitmani*); 6 supralabials, with 3^rd^ and 4^th^ entering orbit (vs. 5 supralabials, with 2^nd^ and 3^rd^ entering orbit in *P.meleagris* and *P.greigerti*); single apical pits on dorsal scales (vs. paired apical pits in *P.ruspolii*); lower number of ventral scales in both sexes (116–129 vs. 153–199 in *P.frontalis*); dorsum with dark black spots (vs. scarlet head and dark body in *P.ornatissima*; uniform dark brown to grey in *P.ambigua* and *P.stuhlmanni*). It further differs from its closest congener, *P.angolensis*, in having two post oculars (vs. one), dorsal large black blotches mostly fused (vs. mostly small paired dorsal grey to black spots), postorbital bone present (vs. absent) and by the presence of four to five well-developed palatine teeth (vs. three reduced teeth).

###### Etymology.

The name *lisima* is derived from the locally spoken Luchaze language in the region of the type locality meaning ‘source’. The full phrase used, ‘*Lisima Lwa Mwondo*’, is translated as “source of life”. This is a reference to central Angola, a high rainfall area where some of the most important rivers in Angola arise. This water makes it its way to the Okavango Delta, sustaining wildlife and local communities in Angola, Namibia and Botswana.

###### Description of holotype

**(Fig. 11).** See Table 4 for further details and meristic data for the holotype. The body is cylindrical and elongated, tapering gradually to a very short tail, 13% total length, tail tip with a prominent spike. Dorsal scales smooth with single apical pits (some suprasubcaudal scales have two apical pits) in 17-15-15 scale rows, scale row reduction from 17 to 16 take place at ventral number 17 with the fusion of 3^rd^ and 4^th^ dorsal scale rows on left side and from 16 to 15 at ventral 23 with the fusion of 3^rd^ and 4^th^ dorsal scale rows on right side; 122 ventral scales; cloaca entire; 13 paired subcaudal scales. Head in dorsal view (Fig. 11B): head narrow and rounded, barely wider than ‘neck’; rostral clearly visible from above, much broader than long (3.39 × 1.06 mm); a single narrow internasal, which is much longer than wide (2.73 × 0.67 mm) and in broad contact with the rostral anteriorly, posteriorly in broad contact with prefrontal and laterally with nasals; single band-like prefrontal which is longer than wide (3.70 × 1.30 mm), in contact laterally with loreal, and posteriorly with the frontal and supraocular scales; frontal pentangular, almost as long as wide (3.09 × 3.00 mm), nearly equal distance to snout (3.30 mm), shorter than prefrontals (3.0 vs. 3.70 mm), but nearly equal in length to the parietal scales (3.00 vs. 3.04 mm), in contact laterally with narrow supraoculars, and posteriorly with two very large parietals; paired parietals as wide as long (3.04 × 3.04 mm), in contact posteriorly with each other and laterally with temporals. Head in ventral view (Fig. 11C): rostral clearly visible from below, protruding well past jawline; mental small, triangular; infralabials seven, first three in contact with single paired chin shields, 1^st^ infralabials in contact with each other; additional three or four rows of smaller gular scales present before start of ventral scales. Head in lateral view (Fig. 11D): snout sharply pointed, longer than the horizontal diameter of eye (ED/SL = 0.49); rostral large with acutely horizontal angular edge, excavated below; nostril is oval shaped, piercing a fully-divided nasal, and directed backwards; nasal scale longer than wide, with anterior part in full contact with rostral, posterior lower corner in contact with 1^st^ supralabial and above with internasal scale and prefrontal; nasal suture present and intersecting 1^st^ supralabial in uppermost corner; single small loreal as long as wide (0.7 × 0.7 mm), in contact below with 1^st^ and 2^nd^ supralabial, above with prefrontal, anteriorly with nasal and posteriorly with single preocular; a single preocular on the right side and two on the left side in contact anteriorly with loreal and prefrontal and above with supraocular, posteriorly protruding of loreal overlap with preoculars to create a small flap; eye large 19.36% headlight, vertical diameter (1.47 mm) two thirds as deep as distance between eye and lip (0.99 mm); pupil round; two postoculars, the lower one largest and in contact with 4^th^ and 5^th^ supralabials, first temporal scale and parietal, the upper smaller in contact with both supraocular and parietal; temporals 1+2 on both sides; narrow elongated supraocular in contact anteriorly with preocular, posteriorly with upper postocular and parietal and above with frontal; six supralabials, 3^rd^ and 4^th^ contacting eye, 5^th^ and 6^th^ supralabial the largest.

**Table 4.** Morphological features and measurements for the type series of *Prosymnalisima* sp. nov. (SVL = snout-vent length, t = truncated).

Catalogue number	PEM R23512	PEM R23456	PEM R23457	PEM R23458	PEM R23483	PEM R23510	PEM R23511	PEM R27381
Type status	Holotype	Paratype	Paratype	Paratype	Paratype	Paratype	Paratype	Paratype
Sex	Male	Female	Male	Male	Male	Male	Female	Male
SVL+TL = total length mm	193+28.9 = 221.9	200+19.3 = 219.3	194+25.8 = 219.8	198+24.6 = 222.6	181+23.8 = 204.8	186+26.3 = 212.3	168+20.6 = 188.6	138+17t = 155
TL/total length ratio (%)	13.0	8.8	11.7	11.1	11.6	12.4	10.9	11.0
Midbody scale rows	17-17-15	17-17-15	17-15-15	17-17-15	17-15-15	17-17-15	17-15-15	17-15-15
Ventral scales	122	121	117	124	121	116	117	122
Subcaudal scales	26	18	22	23	22	23	24	21t
Cloacal Scale	Entire	Entire	Entire	Entire	Entire	Entire	Entire	Entire
Preoculars	1/2	1	2	1	1	2	1	1
Postoculars	2	2	2	2	2	2	2	2
Temporals	1+2	1+2	1+2	1+2	1+2	1+2	1+2	1+2+3
Supralabials (contacting eye)	6 (3,4)	6 (3,4)	6 (3,4)	6 (3,4)	5 (2,3)/6 (3,4)	6 (3,4)	6 (3,4)	6 (3,4)
Infralabials (in contact with 1^st^ chin shield)	7 (3)	7 (3)	7 (3)	7 (3)	7 (3)	7 (3)	7 (3)	7 (3)
Loreal	Yes	Yes	Yes	Yes	Yes	Yes	Yes	Yes
Dorsal colouration	26 fused blotches	30 fused blotches	21 fused blotches	31 fused blotches	22 fused blotches	26 fused blotches	27 fused blotches	36 paired blotches

***Colouration*.** In life (Figs 5C, 11B–D). Dorsum bright yellow-brown with 27 irregular fused black blotches that extend along the back from the nape onto the tail. Each dorsal scale has a darker edge giving it a faint reticulated pattern. Dorsolaterally, between the black vertebral blotches, there is a cluster of 2–4 scales with black edges. The large black nape blotch originates at the posterior margin of the frontal and runs through the parietal onto the dorsal scales, and is approximately nine scale rows deep and eleven scale rows wide. Each vertebral black blotch varies from 4–8 scale rows deep and 3–7 scale row wide. Some of the blotches are fused to form a continuous zig-zag pattern. Frontal and prefrontal sutures have a dark edge forming a pale grey crossbar. Eyes black. Ventrum cream-white, with the two outermost dorsal scale rows same colour as ventrum. In preservative (Fig. 11A). Same as in life, but yellow-brown colouration faded and the dark edges became more noticeable. Ventrum beige.

***Paratype and additional material variation*.** See Table 2 and 4 for full meristic data. Dorsal scales smooth and in 17-17-15 rows at midbody; 116–124 (116–124 males, 117–129 females) smooth ventral scales; 18–26 (22–26 males, 18–24 females) paired subcaudal scales; one (rarely two) preoculars; two postoculars; temporals mostly 1+2; mostly six supralabials, with 3^rd^ and 4^th^ entering the orbit; seven infralabials, with first three in contact with the chin shield, cloacal scale entire; 21–36 fused dark dorsal spots. Largest female: 275+28 mm (NMZ UM 21272: 15 km WSW of Katima Mulilo); largest male: 198+25 mm (PEM R23458: Quembo River source). The colouration of the type material is in general in agreement with the holotype, except that the dorsal fused blotches vary in size, number and arrangement (Fig. 5A–C). The nape black blotch always originates at the anterior part of the frontal extending through the parietals to 7–9 dorsal scale rows deep, 11–15 scales wide and start from the 3^rd^–5^th^ lateral dorsal scale row. The dorsum consists of 21–36 confluent black blotches that are 7–11 scales wide and three to four scales deep. One specimen (PEM R23456) exhibits a distinct dark interorbital band and internasal band. The only juvenile collected (PEM R27381, Fig. 5C) has small paired black blotches (two scales deep and four scales wide) on a lighter yellow ground colour, large head blotch starts at posterior frontal through parietals, seven scales wide.

***Skull osteology and teeth* (Figs 7–9).** This species presents a compact and rigid skull, common among *Prosymna* species with unfused braincase and nasal bones. Parietals are fused. Postorbital bone is present and contributes to the posterior edge of the orbital rim. Premaxilla has a short but robust ascending nasal process that lies between the ventral laminae of the nasals with low profile of the anterior portion which gradually slope ending in a moderate narrow tip and two elongated maxillary processes. Maxilla and premaxilla are in contact. Nasal bones are medium large bones in contact with frontal and premaxilla. Septomaxilla is a well-developed bone, in broad contact with the premaxilla, frontal, vomer, prefrontal and frontal bones. Vomer is well developed with a perforated dorsolateral portion. Maxillary is reduced anteriorly with an elongated pick-shaped palatine process with five or six laterally reduced curved tooth loci, followed by four to five enlarged lancet-shaped tooth loci, on same disposition. Palatine with four to five well developed teeth and an enlarged dorsal curved vomerine process. Pterygoid is a thin elongated bone. Supratemporal is in broad contact with the quadrate and participates in the lateral movement of the lower jaw. The lower jaw presents a compound bone, splenial, coronoid and dentary. Coronoid and splenial are reduced, almost vestigial. Dentary with eight tooth loci.

***Hemipenis*.** Short simple structure, only reaching the 6–9^th^ ventral scale. Single non-bifurcated sulcus. Ornamentation is flounced. Proximal third is smooth. Distal portion with four to five flounces that starts at the sulcal fold and encircle the whole organ, the most proximal often branched, forming a pocket of which the edges is smooth, tapering into a distal point. Retractor muscle is straight.

###### Natural history notes.

All specimens were caught in late November during the rainy season. At this time, many adult lacertids, *Ichnotropiscapensis* and I.cf.grandiceps, were also observed mating in the same habitat. *Prosymna* are well known to prey on soft-shell lizard eggs, and *P.lisima* sp. nov. may actively seek out these lacertids’ eggs. Only two of the females had stomach contents, while all the males had empty stomachs. The largest female (PEM R23456) had three empty lizard egg shells in the hind gut, three empty egg shells at the rear end of the stomach, and four undigested lizard eggs in the main stomach (Fig. 12). Another female (PEM R23511) had four undigested lizard eggs in the main stomach. All eggs measured ~ 11.0 mm in length and each had a lateral cut. The eggs in the main stomach also all had a lateral cut but still maintained their shape and were surrounded by calcified leaked yolk (due to the preservation process). The eggs at the rear and the hindgut were all undecomposed and compressed. One of the paratype females (PEM R23456) was gravid, and had three eggs in early developmental stages (16.8 × 4.2 mm). Interestingly, on two different occasions, three specimens (two males and one female) were caught on the same night in the same trap array. This may indicate that males were following females to breed.

###### Distribution and habitat.

Currently only known from east-central Angola, western Zambia and the Zambezi Region of north-eastern Namibia. In the region of Katima Mulilo (eastern Zambezi Region, Namibia) it occurs in sympatry with its sister species, *P.angolensis* (Fig. 2). The tentative assigned material from Kuvango (Monard 1931) needs verification, but it agrees in colouration and morphology to the new species. This species is expected to be much more widely distributed in the Kalahari basin, as it seems to be associated with the deep Kalahari sands. The Angolan material occurs in Angolan moist miombo woodland, while the Zambian and Namibian material occurs in dry miombo woodland. The elevation ranges between 950 and 1450 m a.s.l. All newly collected specimens were captured in trap arrays set in sandy areas next to river source lakes or main rivers in eastern Angola (Conradie et al. 2021).

##### Prosymna confusa sp. nov.

Taxon classificationAnimaliaSquamataProsymnidae

08424C0A-45F1-5E4F-ABB8-0A90FAEC1D6A

https://zoobank.org/A4E2E3E8-A658-4007-B5F4-46D875457EA1

**Figure 13.**
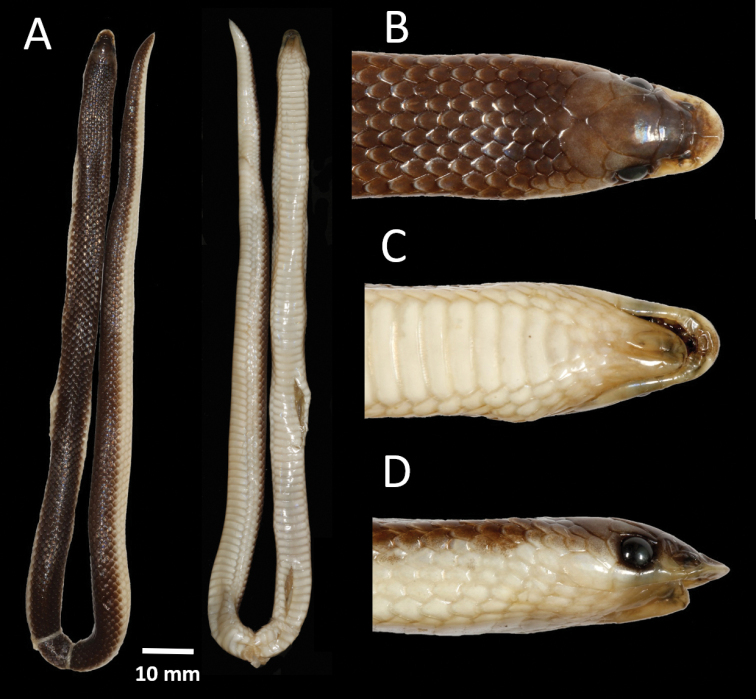
Holotype of *Prosymnaconfusa* sp. nov. (PEM R24013) from 20 km west of Lola on the road northwest to Camacuio and on the edge of Bentiaba River, Namibe Province, Angola **A** dorsal and ventral full body **B** dorsal head **C** ventral head **D** lateral head.

Figs 6 , 7 , 8 , 9 , 13 Common names: Plain Shovel-snout Snake (English); Cobra-de-focinho-de-pá-lisa (Portuguese).

###### Chresonymy.

*Prosymnaangolensis*: Bogert 1940: 59; Monard 1937: 123 (in part); Broadley 1980: 152 (in part).

*Prosymnaambigua*: Branch 2018: 64, fig. 24; Pietersen et al. 2021: 96, fig.

Monard (1937) was the first to document a uniformly grey specimen from Ebanga. This was followed by Bogert (1940) who documented a specimen from Capelongo (AMNH R50504) that also exhibited a uniform pale brown dorsum with small white spots (similar to *P.meleagris* pattern). This uniform dorsum colouration is in agreement with the new specimen collected from coastal Angola (Branch 2018) and this colouration is very distinct from the other two species, yellowish grey with paired small black dorsum spots in *P.angolensis* and bright yellow with fused black blotches in *P.lisima* sp. nov.

###### Material examined.

***Holotype*** (female). PEM R24013, collected from 20 km west of Lola on the road northwest to Camacuio, on the edge of Bentiaba River (-14.27583, 13.45806, 791 m a.s.l.), Namibe Province, Angola by William R. Branch, Pedro Vaz Pinto and João S. de Almeida on 2 November 2015.

###### Additional material tentatively assigned to the new species.

AMNH 50504, Capelongo, approx. -14.46645, 16.29241, Huíla Province, Angola (Bogert 1940: 59); Ebanga, approx. -12.73333, 14.73333 , Benguela Province, Angola (Monard 1937: 123).

###### Diagnosis.

The new species differs from other *Prosymna* species in the following characters: rostral sharply depressed and angular (vs. rounded in *P.visseri*); presence of a single band-like internasal (vs. paired internasals in *P.somalica*, *P.bivittata*, *P.sundevalli*, *P.lineata*); dorsal scales smooth (keeled in *P.janii*); midbody scale rows 15–17 (vs. 19–21 in *P.pitmani*); six supralabials, with 3^rd^ and 4^th^ entering orbit (vs. five supralabials, with 2^nd^ and 3^rd^ entering orbit in *P.meleagris* and *P.greigerti*); single apical pits on dorsal scales (vs. paired apical pits in *P.ruspolii*); lower number of ventral scales in both sexes (116–129 vs. 153–199 in *P.frontalis*); dorsum uniform dark grey (vs. scarlet head and dark body in *P.ornatissima*). It further differs from its closest congeners in the *angolensis* group: one postocular (vs. two in *P.lisima* sp. nov.), dorsum uniform grey (vs. dorsum with large mostly fused black blotches in *P.lisima* sp. nov. and mostly smaller paired longitudinal rows of grey to black spots in *P.angolensis*), postorbital bone absence (vs. present in *P.lisima* sp. nov.), presence of two well-develop palatine teeth (vs. four to five in *P.lisima* sp. nov. and three reduced teeth in *P.angolensis*), fused braincase (vs. unfused in *P.angolensis* and *P.lisima* sp. nov.) and two frontal foramina (vs. three to four in *P.angolensis* and *P.lisima* sp. nov.).

###### Etymology.

When the late Bill Branch collected the holotype, he was unsure of its identification and referred to it as an unusual specimen that could not be assigned to any known species from Angola. He later referred to it as *P.ambigua* (Branch 2018), presumably based on its uniform grey colouration. The name *confusa* is a reflection of the confusion this specimen has caused and of the general confusion in the *P.angolensis* group.

###### Description of holotype

**(Fig. 13).** Adult female measuring 240 mm SVL+29 mm TL = 269 mm total length. The body is cylindrical and elongated, tapering gradually to a very short tail, 10.8% total length, tail tip with prominent spike. Dorsal scales smooth, with single apical pits, in 17-15-15 scale rows, scale row reduction from 17 to 16 take place at ventral number 29 with the fusion of 3^rd^ and 4^th^ dorsal scale row on right side and from 16 to 15 at ventral 32 with the fusion of 3^rd^ and 4^th^ dorsal scale row on left side; 151 ventral scales; cloaca entire; 26 subcaudal scales. Head in dorsal view (Fig. 13B): head narrow and rounded, barely wider than ‘neck’; rostral clearly visible from above, much broader than long (2.84 × 1.47 mm); a single narrow internasal, which is much longer than wide (2.40 × 0.57 mm) and in broad contact with the rostral anteriorly, posteriorly in broad contact with prefrontal and laterally with nasals; single band-like prefrontal which is longer than wide (3.71 × 1.25 mm), in contact laterally with loreal and posteriorly with the frontal and supraocular scales; frontal pentangular, almost as long as wide (2.97 × 3.14 mm), nearly equal in length to the distance to snout (2.91 mm), more than double than prefrontal width (3.14 vs. 1.25 mm), and three quarters the length of the parietals (3.14 vs. 2.43 mm), in contact laterally with narrow supraoculars, and posteriorly with two very large parietals; paired parietals longer than wide (2.28 × 2.43 mm), in contact posteriorly with each other and laterally with temporals. Head in ventral view (Fig. 13C): rostral clearly visible from below, protruding well past jawline; mental small, triangular; infralabials eight on right side and nine on left side, first three in contact with single paired chin shields, 1^st^ infralabials in contact with each other; additional three rows of smaller gular scales present before the start of ventral scales. Head in lateral view (Fig. 13D): snout sharply pointed, longer than the horizontal diameter of eye (ED/SL = 0.45); rostral large with acutely horizontal angular edge, excavated below; nostril is oval shaped, piercing divided nasal, and directed backwards; nasal scale longer than wide, with anterior part in full contact with rostral, posterior lower corner in contact with 1^st^ supralabial, upper section in contact with internasal scale and prefrontal and posteriorly with loreal and prefrontal; nasal suture present and intersecting loreal; single small loreal as long as wide (0.84 × 0.84 mm), in contact below with 1^st^ and 2^nd^ supralabial, above with prefrontal, anteriorly with nasal and posteriorly with single preocular; single preocular on both sides in contact anteriorly with loreal and above with supraocular and prefrontal, posteriorly with loreal; eye large 16.50% HL, vertical diameter (1.53 mm), two thirds as deep as distance between eye and lip (0.42); pupil round; one postoculars, in contact with 4^th^ upperlabial, 1^st^ temporal scale, supraocular, and parietal; temporals 1+2; narrow elongated supraocular in contact anteriorly with preocular and prefrontal, posteriorly with the postocular and above with frontal; five supralabials on both sides with 3^rd^ and 4^th^ in contact with eye on right and 2^nd^, 3^rd^ and 4^th^ on left, 5^th^ and 6^th^ supralabial the largest.

***Colouration*.** In life (Fig. 6). Dorsum uniform grey with the anterior edges of scales with a white spot, outermost two to three scale rows white, with only the first outermost scale row of tail white. Nape with a faint collar that is three scale rows wide. The prefrontal and internasal black compared to the rest of the head being grey. Eye black. Ventrum white. In preservative (Fig. 13). Same as in life, but the grey faded and became brown. Ventrum beige.

**Figure 14.**
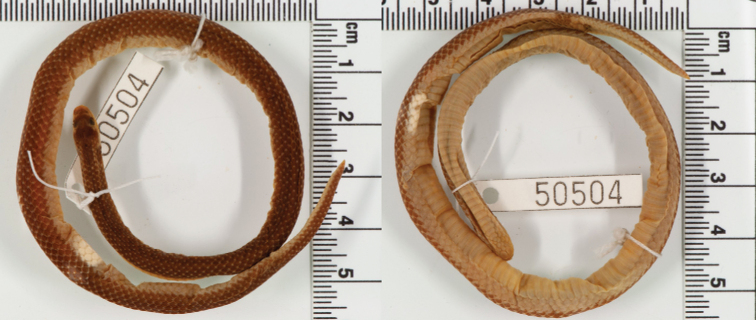
A specimen (AMNH 50504) form Capelongo, approx. -14.46645, 16.29241; Huíla Province, Angola, assignable to the new species, *P.confusa* sp. nov. (Photos: Lauren Vonnahme, American Museum of Natural History).

***Additional material variation*.** See Table 2 for summarised meristic data. Only data of three females were available, but the assignment of historical material to *P.confusa* sp. nov. still requires confirmation. Dorsal scales smooth and in 17-17-15 rows at midbody; 143–155 smooth ventral scales; 17–26 paired subcaudal scales; one preoculars; one postoculars; temporals 1+2; five or six supralabials, with 3^rd^ and 4^th^ entering the orbit; seven infralabials, with first three in contact with the 1^st^ chin shield, cloacal scale entire. Largest female: 240+29 mm (holotype PEM R24013). The colouration is similar to the holotype, except that in the Capelongo specimen (AMNH R50504) the white spots are much more conspicuous (Fig. 14). The specimen from Ebanga is unaccounted for (Broadley 1980), but Monard (1937) described the colouration as uniform grey above.

***Skull osteology and teeth* (Figs 7–9).** The holotype presents a compact and rigid skull common among *Prosymna* species with fused braincase, fused parietal, and unfused frontal and nasal bones. Postorbital bone is absent. Parietal with a fronto-lateral sharp edge that participates virtually as posterior edge of the orbital rim. Premaxilla has a well-developed and robust ascending nasal process that lies between the ventral laminae of the nasals with low profile of the anterior portion which gradually slope to a narrow tip and two elongated maxillary process in contact with the maxilla. Nasal bones are medium large bones in contact with frontal and premaxilla. Septomaxilla is a well-developed bone, in broad contact with premaxilla, frontal, vomer, prefrontal and frontal bone. The vomer is well developed with performed dorsolateral portion of the bone. Maxillary is reduced anteriorly with an elongated pick-shaped palatine process with six to seven laterally reduced curved tooth loci, followed by four to five enlarged lancet-shaped tooth loci. Palatine with two well developed teeth and an enlarged dorsal and curved vomerine process. Pterygoid is a thin elongated bone. Supratemporal is in broad contact with the quadrate and participates in the lateral movement of the lower jaw. The lower jaw is comprised of compound, splenial, coronoid, and dentary bones. Coronoid and splenial bones are reduced, almost vestigial. Dentary bone with six tooth loci.

***Hemipenis*.** Unknown. Bogert (1940) suggested it to be similar to *P.ambigua*. However, *Prosymnaambigua* is unique in having a very long ‘telescopic’ hemipenis that is longer than the tail, which is not present in the *sundevalli* group (Broadley 1980). The latter group, to which *P.confusa* sp. nov. belongs, is characterised by its short hemipenes (8–10^th^ ventral scales long vs. longer than tail in *P.ambigua*), low number of flounces (5–6 vs. more than 50 in *P.ambigua*) and straight retractor muscle (telescopic in *P.ambigua*). *Prosymnafrontalis* shares a similar hemipenile structure with *P.ambigua*.

###### Natural history notes.

The holotype was found actively moving around near a large rock outcrop during the day.

###### Distribution and habitat.

This species is endemic to southwestern Angola (Fig. 2), and appears to be associated with mopane woodlands, dry savannas, and semi-desert shrublands (Barbosa 1970). The new specimen was found in sandy plains with scattered low granite outcrops, with varying degrees of short grass cover and scattered bushes. Vegetation included *Colophospermummopane*, *Ficus* sp., *Senegalia* (= *Acacia*) *mellifera*, *Commiphora* sp., *Bosciafoetida*, and *Salvadorapersica*. The two additional historical specimens from Ebanga and Capelongo that are tentatively assigned to this species occurred in similar dry habitat.

## Discussion

The taxonomic status of *Prosymnaangolensis* has been the subject of debate by several researchers over time. Thanks to the access to new material, we are able to describe two new species and provide the first phylogenetic placement of *P.angolensis*. Our work recovered a similar topology to that of Heinicke et al. (2020), with four clades characterising Prosymnidae. The addition of the new Angolan samples did not help resolve the alpha taxonomy or provide better support for deeper relationships between and within groups, as in Heinicke et al. (2020). However, our sampling does allow for a broader understanding of the phylogenetic relationships in the *sundevalli* group.

While the phylogenetic placement between lineages within the *angolensis* subgroup remains unresolved (particularly *P.angolensis* sensu stricto) and species pairwise distances are below those of their congeners; the topological support offered by ML and BEAST coupled with species delimitation analyses lends support for the distinctiveness of the two novel taxa at a genetic level. A single, near topotypical, sample of *P.angolensis* was available for molecular analysis and, based on the disjunct distribution between the populations of Angola and Namibia, Botswana, and Zimbabwe of *P.angolensis*, cryptic diversity is expected and worth further investigation.

Whilst our phylogeny lacks the resolution necessary to resolve the inter-specific relationships within the *sundevalli* group, which is likely a product of incomplete taxon sampling, it is clear that the *angolensis* subgroup is within the larger *sundevalli* group, as suggested by Broadley (1980). Our work further identified two new lineages within the *angolensis* subgroup, one of which agrees with differences in morphology and colouration previously reported from western Zambia and the Zambezi Region (Broadley 1980). Although the ‘eastern race’ was partly defined based on the lower ventral scale counts (Broadley 1980), we show here that the differences are not significant and that these might be related to the sizes of the animals collected. Studies have shown positive correlation between ventral and subcaudal scales and body size, and that caution should be taken when used as a diagnostic feature (Lindell 1994; Lee et al. 2016). Based on the clear morphological (head scalation and osteology), dorsal colouration, and genetic differences reported in this study, we here describe these two lineages as *P.lisima* sp. nov. and *P.confusa* sp. nov. This raises the total number of *Prosymna* species to 18, with six species occurring in Angola. This number is, however, expected to grow as further cryptic diversity has been identified in *P.frontalis*, *P.ambigua*, and *P.stuhlmanni* (Heinicke et al. 2020) that may warrant taxonomic re-evaluation.

Because we only compared a small series of skulls for the osteological characterisation (one or two scans per species), further intra-specific variation may exist, thus the osteological differences observed should be taken with caution. That said, there were some striking differences among the groups, as mentioned by Heinicke et al. (2020). The postorbital was only recorded in *P.ambigua* and *P.stuhlmanni* and suggested as vestigial in *P.greigerti* by Heinicke et al. (2020). Here we confirm the presence of a well-developed postorbital bone in *P.janii*, the only remaining member of the *ambigua* group. Additionally, we recorded the presence of a postorbital bone in two additional species, *P.lisima* sp. nov. and P.cf.frontalis, being almost absent in the latter. This shows that the postorbital bone development is not restricted to the *ambigua* group but rather, present in the south-western taxa and the *sundevalli* group. This bone was present at a well-developed state in the *ambigua* group (reaching the maxilla) and *P.lisima* sp. nov. (slightly reduced, only reaching midway to the maxilla), but being small, almost absent in P.cf.frontalis. The purpose of the postorbital bone is currently unknown, but it may be associated with additional muscle attachment points to aid in feeding or crushing lizard eggs. This may suggest that the postorbital bone has been lost several times from its ancestral trait as a consequence of diet specialisation. Therefore, we recommend further research along this line, to shed light on the evolutionary history of this group.

In Prosymnacf.frontalis, the anterior portion of the maxilla has no teeth, similar to that in *P.visseri*. Heinicke et al. (2020) speculated, based on the fact that *P.frontalis* is also more rupiculous than its fossorial relatives, they may also have lost these anterior maxillae to allow them to feed on more hard-shelled gecko eggs which are found sympatrically. Although *P.confusa* sp. nov. may occur in similar habitat to P.cf.frontalis, it does not share the same maxillary tooth arrangement, which may indicate a different feeding strategy, such as feeding on sympatrically occurring soft-shelled lacertid eggs (e.g., *Heliobolus* sp. and *Pedioplanis* sp.). *Prosymnaconfusa* sp. nov. shares similar dentary development to P.cf.frontalis, in that the most anterior section of the dentary bone is free of teeth, compared to other species where they extend almost to the tip of the dentary.

The descriptions of two new species, *P.confusa* sp. nov., endemic from dry habitats in southwestern Angola, and *P.lisima* sp. nov., associated to the Kalahari sands from Angola to neighbouring countries to the east, are an indication of how much diversity is likely still to be described from these regions. In the last decade a renewed interest in the Angolan herpetofauna has led to numerous expeditions to remote areas. Consequently, the number of species recorded from the country, has increased considerably (Conradie et al. 2012a, 2012b, 2013, 2020; Stanley et al. 2016; Ceríaco et al. 2018, 2020a, 2020b, 2020c, 2021b; Branch et al. 2019, 2021; Marques et al. 2019a, 2019b, 2020, 2022a, 2022b; Hallermann et al. 2020; Nielsen et al. 2020; Baptista et al. 2021; Lobón-Rovira et al. 2021; Parrinha et al. 2021; Wagner et al. 2021). Among these are four new species of snakes (Conradie et al. 2020; Hallermann et al. 2020). The addition of two new species of *Prosymna* brings the number of recorded snake species from Angola to ca. 135. This is approximately 69% of the known squamate diversity recorded from the country. As more research is conducted on Angolan herpetofauna, many more new species descriptions and additions are expected. Finally, we raise the importance of further surveys in this poorly studied region of Africa, with the aim of collecting recent material that will allow us to clarify the taxonomic placement of several species’ complexes and poorly understood species.

## Supplementary Material

XML Treatment for Prosymna angolensis

XML Treatment for Prosymna lisima

XML Treatment for Prosymna confusa

## Footnotes

Broadley (1980) listed Werner (1929: 142) as chersonomy of Prosymna angolensis, but we have removed it in this work as it refers to Psammophis angolensis (Bocage, 1872).

## Appendix 1

**Table A1.** List of samples used in this study and their associated metadata. NA–Not Available.

Species	Voucher ID	Locality	Molecular Markers					
			*16S*	*ND2*	*cyt-b*	*c-mos*	*RAG1*	*ENC-1*
**Ingroup**								
*Prosymnaangolensis*	NB 0521/CHL 0521	Bicuar National Park, Huíla, Angola	OP288036	OP289543	OP289533	OP289553	NA	NA
*P.confusa* sp. nov. ‘Coastal’	AG 014/PEM R24013	20 km W Lola, road northwest to Camacuio, Namibe, Angola	OP288044	OP289552	OP289542	OP289562	NA	NA
*P.lisima* sp. nov. ‘Eastern’	WC-4694/PEM R23456	Quembo River Source, Moxico, Angola	OP288037	OP289544	OP289534	OP289554	NA	NA
*P.lisima* sp. nov. ‘Eastern’	WC-4696/PEM R23457	Quembo River Source, Moxico, Angola	OP288038	OP289545	OP289535	OP289555	NA	NA
*P.lisima* sp. nov. ‘Eastern’	WC-4706/PEM R23458	Quembo River Source, Moxico, Angola	OP288039	OP289546	OP289536	OP289556	NA	NA
*P.lisima* sp. nov. ‘Eastern’	WC-4810/PEM R23483	Cuando River Source, Moxico, Angola	OP288040	OP289547	OP289537	OP289557	NA	NA
*P.lisima* sp. nov. ‘Eastern’	WC-4870/PEM R23510	Cuito Source Lake, Moxico, Angola	OP288041	OP289548	OP289538	OP289558	NA	NA
*P.lisima* sp. nov. ‘Eastern’	WC-4863/PEM R23511	Cuito Source Lake, Moxico, Angola	OP288042	OP289549	OP289539	OP289559	NA	NA
*P.lisima* sp. nov. ‘Eastern’	WC-4862/PEM R23512	Cuito Source Lake, Moxico, Angola	OP288043	OP289550	OP289540	OP289560	NA	NA
*P.lisima* sp. nov. ‘Eastern’	WC-6844/PEM R27381	Quembo River bridge camp, Moxico, Angola	NA	OP289551	OP289541	OP289561	NA	NA
*P.ambigua*	CAS 258670	Cangandala NP, Malanje, Angola	MT453136	MT460633	NA	MT460596	MT460655	NA
*P.ambigua*	GJ 2762	Malanje, Angola	MT453137	MT460634	NA	MT460597	MT460656	NA
*P.ambigua*	PEM R16763	Gnimeti River, Klein’s Camp, Loliondo Game Controlled Area, Mara, Tanzania	MT453112	NA	NA	MT460579	MT460635	NA
*P.bivittata*	AMB (53F6)	NA	MT453113	MT460610	NA	NA	NA	NA
*P.bivittata*	PEM R17431	Mkhuze Falls Private Game Reserve, KwaZulu-Natal, South Africa	MT453114	MT460611	NA	NA	MT460636	NA
*P.bivittata*	PEM R17432	Mkhuze Falls Private Game Reserve, KwaZulu-Natal, South Africa	MT453115	MT460612	MT482412	MT460580	MT460637	MT460598
*P.bivittata*	PEM R17433	Mkhuze Falls Private Game Reserve, KwaZulu-Natal, South Africa	MT453116	MT460613	MT482413	MT460581	MT460638	NA
P.cf.frontalis	MCZ R193166	Farm Omandumba, Erongo, Namibia	MT453117	MT460614	NA	NA	MT460639	NA
P.cf.frontalis	PEM R17996	Espinheira, Namibe, Angola	MT453118	MT460615	MT482414	MT460582	MT460640	MT460599
P.cf.frontalis	MCZ R184857	Hobatere Lodge, Namibia	MT453119	NA	NA	NA	MT460641	NA
*P.frontalis*	MCZ R185112	Farm Oas, Karas, Namibia	MT453120	MT460616	NA	NA	NA	NA
*P.frontalis*	MCZ R185003	Geister Schlucht, Klein Aus Vista, Karas, Namibia	MT453121	MT460617	KR814693	KR814680	MT460642	
*P.greigerti*	E 107.19	NA	JF340124	NA	NA	NA	NA	NA
*P.greigerti*	E 124.1	NA	JF340125	NA	NA	NA	NA	NA
*P.greigerti*	UTEP 22161	Pian Upe Wildlife Reserve, Hyena Hill, Northern Region, Uganda	NA	MT460632	NA	MT460595	MT460654	NA
*P.janii*	JM 1045	KwaZulu-Natal, South Africa	MT453122	MT460618	MT482415	NA	MT460643	NA
*P.janii*	MCZ Z37878	KwaZulu-Natal, South Africa	MT453123	NA	NA	NA	NA	NA
*P.janii*	AMB (SNH8)	KwaZulu-Natal, South Africa	MT453124	MT460620	MT482417	MT460583	MT460644	MT460601
*P.janii*	PEM R12072	Madlangula, Kosi Bay Nature Reserve, KwaZulu-Natal, South Africa	FJ404222	NA	FJ404319	FJ404293	NA	NA
*P.janii*	PEM R17372	uMkhuze, Greater St. Lucia Wetland Park, KwaZulu-Natal, South Africa	NA	MT460619	MT482416	NA	NA	MT460600
*P.lineata*	MCZ R184472	7.5 km E of Musina on Tishipe Rd., Limpopo, South Africa	MT453126	MT460622	MT482419	MT460585	MT460646	MT460602
*P.lineata*	JM 1816	Khamai, Limpopo, South Africa	MT453127	MT460623	NA	MT460586	MT460647	NA
*P.lineata*	MBUR 394	Makgabeng area, Limpopo, South Africa	MT453128	MT460624	MT482420	NA	NA	MT460603
*P.lineata*	AMB (53F8)	Blouberg, Limpopo, South Africa	MT453125	MT460621	MT482418	MT460584	MT460645	NA
*P.meleagris*	E 107.22	NA	JF340122	NA	NA	NA	NA	NA
*P.meleagris*	E 113.2	NA	JF340123	NA	NA	NA	NA	NA
*P.meleagris*	MVZ245380	Shai Hills, Greater Accra Region, Ghana	MT453135	MT460631	NA	MT460594	MT460653	NA
*P.ruspolii*	CMRK316	Tanzania	NA	NA	DQ486347	DQ486171	NA	NA
*P.stuhlmanni*	LHM-000270	3.2 km W of Lesheba Wilderness Reserve, Limpopo, South Africa	NA	MT460625	MT482421	MT460587	NA	NA
*P.stuhlmanni*	PEM R17402	Mkhuze Game Reserve, KwaZulu-Natal, South Africa	MT453129	MT460626	MT482422	MT460588	MT460648	MT460604
*P.sundevalli*	MCZ R184401	Farm Newstead, Eastern Cape, South Africa	MT453130	MT460627	MT482423	MT460589	MT460649	MT460605
*P.sundevalli*	MCZ R184512	23.9 km NE Vaalwater on Rd. to Melkrivier, Limpopo, South Africa	MT453131	MT460628	MT482424	MT460590	MT460650	MT460606
*P.visseri*	CAS 214753	Opuwo Dist. ca 2 km N of Sesfontein, Kunene Region, Namibia	MT453132	MT460629	MT482425	MT460591	MT460651	MT460607
*P.visseri*	PEM R17994	Espinheira, Namibe, Angola	MT453133	MT460630	MT482426	MT460592	MT460652	MT460608
*P.visseri*	MG 302	Farm Kaross, Kunene Region, Namibia	MT453134	NA	MT482427	MT460593	NA	MT460609
**Outgroup**								
*Psammophiscondanarus*	NA	NA	Z46479	AY058991	AF471075	AF471104	NA	NA
*Pseudaspiscana*	NA	NA	AY611898	AY058992	AY612080	AY611989	NA	NA
*Aparallactuswerneri*	NA	NA	AY188045	NA	AF471035	AF471116	NA	JN881241
*Atractaspiscorpulenta*	NA	NA	AY611837	NA	AY612020	AY611929	NA	JN881242
*Boaedonfuliginosus*	NA	NA	KX249802	NA	KM519712	AF544686	KM519725	JN881267
*Buhomaprocterae*	NA	NA	AY611818	NA	AY612001	AY611910	NA	NA
*Lampropeltisgetula*	NA	NA	KX694649	MG672874	MG672798	KX694811	MG673016	JN881266
*Leioheterodonmadagascariensis*	NA	NA	AY188061	AY059010	AY188022	AF544685	NA	NA
*Najakaouthia*	NA	NA	KX277260	AY059008	AF217835	AY058938	JF412633	JN881273
*Oxyrhabdiumleporinum*			NA	NA	AF471029	DQ112081	NA	NA

**Table A2.** High Resolution X-ray Computed Tomography (HRCT) parameters used to scan *Prosymna* skulls.

Species	Catalogue Number	Voxel (mm)	Voltage (kV)	Current (μA)	Exposure Time (secs)	MorphoSource
*Prosymnaangolensis*	SAM ZR16574	0.00499991	60	300	0.5	https://doi.org/10.17602/M2/M435305
*Prosymnaconfusa* sp. nov.	PEM R24013	0.00799991	90	100	0.5	https://doi.org/10.17602/M2/M435274
*Prosymnalisima* sp. nov.	PEM R23512	0.00699991	60	300	0.5	https://doi.org/10.17602/M2/M435310
*Prosymnalisima* sp. nov.	PEM R23510	0.00699991	60	300	0.5	https://doi.org/10.17602/M2/M435343
Prosymnacf.frontalis	PEM R17997	0.00699991	100	80	0.5	https://doi.org/10.17602/M2/M435295
*Prosymnajanii*	PEM R08679	0.00699991	90	80	0.5	https://doi.org/10.17602/M2/M435300
